# Glycoproteomic studies of IgE from a novel hyper IgE syndrome linked to PGM3 mutation

**DOI:** 10.1007/s10719-015-9638-y

**Published:** 2015-12-19

**Authors:** Gang Wu, Paul G. Hitchen, Maria Panico, Simon J. North, Mohamed-Ridha Barbouche, Daniel Binet, Howard R. Morris, Anne Dell, Stuart M. Haslam

**Affiliations:** Department of Life Sciences, Imperial College London, South Kensington Campus, London, SW7 2AZ UK; Division of Cell Signalling and Immunology, School of Life Sciences, University of Dundee, Dundee, DD1 5EH UK; Laboratory of Immunopathology, Vaccinology and Molecular Genetics, Pasteur Institute of Tunis and University Tunis El Manar, Tunis, Tunisia; MS-RTC (Mass Spectrometry Research and Training Centre), Suite 3.1 Lido Medical Centre, St. Saviours Road, Jersey, JE2 7LA UK; Department of Life Sciences, Faculty of Natural Sciences, Imperial College London, South Kensington Campus, London, SW7 2AZ UK

**Keywords:** IgE, Glycoproteomics, Hyper IgE Syndrome, Phosphoglucomutase 3

## Abstract

Glycans serve as important regulators of antibody activities and half-lives. IgE is the most heavily glycosylated antibody, but in comparison to other antibodies little is known about its glycan structure function relationships. We therefore describe the site specific IgE glycosylation from a patient with a novel hyper IgE syndrome linked to mutations in PGM3, which is an enzyme involved in synthesizing UDP-GlcNAc, a sugar donor widely required for glycosylation. A two-step method was developed to prepare two IgE samples from less than 1 mL of serum collected from a patient with *PGM3* mutation and a patient with atopic dermatitis as a control subject. Then, a glycoproteomic strategy was used to study the site-specific glycosylation. No glycosylation was found at Asn264, whilst high mannose glycans were only detected at Asn275, tri-antennary glycans were exclusively observed at Asn99 and Asn252, and non-fucosylated complex glycans were detected at Asn99. The results showed similar glycosylation profiles between the two IgE samples. These observations, together with previous knowledge of IgE glycosylation, imply that IgE glycosylation is similarly regulated among healthy control, allergy and PGM3 related hyper IgE syndrome.

**Electronic supplementary material:**

The online version of this article (doi:10.1007/s10719-015-9638-y) contains supplementary material, which is available to authorized users.

## Introduction

All antibodies are glycosylated and the glycans serve as important regulators for antibody activities [1–6] and half-lives [7, 8]. IgE is known to play a key role in allergic responses [9]. It is the most heavily glycosylated antibody and contains seven potential N-glycosylation sites at Asn21, 49, 99, 146, 252, 264 and 275 in the heavy chain constant region. However, glycosylation of IgE is not extensively studied, due to its low concentration in the blood, making it difficult to prepare. In the 1970s, monosaccharide ratios of glycans at each potential glycosylation site on a myeloma IgE were reported [10–12]. High variations of glycosylation among different sites were observed, for example Asn264 was found not to be glycosylated and only Man and GlcNAc residues were detected at Asn275. The other 5 sites were occupied by complex glycans with different levels of fucosylation and sialylation. In 2004, an overall profile of IgE N-glycans from an atopic patient was obtained using HPLC [13], which detected 24 N-glycans including both high mannose glycans and complex glycans. More than 75 % of the glycan pool was sialylated, almost 60 % of glycans were core fucosylated, and 13 % of glycans had bisecting GlcNAc. Recently, a mass spectrometry based glycoproteomic strategy was used to obtain a site specific analysis of IgE N-glycosylation [14]. The results showed for the first time the glycan structures at each site on IgE, which was collected from a hyperimmune donor, from the pooled serum of multiple nondiseased donors, and from the pooled serum of 2 patients with IgE myeloma. Consistent with the monosaccharide studies of a myeloma IgE, the data showed Asn264 was not glycosylated and Asn275 was glycosylated exclusively with high mannose glycans.

However, little is known about the glycosylation of IgE from patients with hyper IgE syndrome (HIES). HIES is a primary immunodeficiency disorder which is characterized by dramatically elevated IgE levels, accompanied by other symptoms such as eczema, recurrent staphylococcal skin infections and pulmonary infections [15]. The disease was first reported in 1966 [16], and its autosomal dominant inheritance pattern was discovered in 1999 [17]. Mutations in two proteins were discovered to be linked to the disease: signal transducer and activator of transcription 3 (STAT3) [18, 19] and dedicator of cytokinesis protein 8 (DOCK8) [20, 21]. Recently, a novel hyper IgE syndrome has been discovered [22, 23], without mutations in STAT3 or DOCK8. Instead, the disease is linked to mutations in phosphoglucomutase 3 (PGM3). Unlike other phosphoglucomutases, which catalyse the conversion of Glc-6-phosphate and Glc-1-phosphate, PGM3 catalyses the conversion of GlcNAc-6-phosphate and GlcNAc-1-phosphate, producing the precursor for synthesizing UDP-GlcNAc, which is a sugar donor required for glycosylation. Our glycomic studies have already found dramatic changes of N-glycans in Epstein–Barr virus (EBV) treated B cells and neutrophils from these patients [23]. Moreover, our glycomic studies of IgE showed that the overall profile of N-glycans did not change significantly from a patient with *PGM3* mutations and a patient with atopic dermatitis [23]. Both IgE samples had high mannose glycans and complex glycans. Most of the complex glycans were bi-antennary with core fucose and sialic acid. Bisecting GlcNAc was observed in some of the bi-antennary structures. Tri-antennary glycans and truncated glycans were also detected. The relative intensities among these glycans were similar between the two IgE samples. However, it is unknown whether there are site specific changes which could be involved in the elevated IgE which could affect IgE activities and half-life.

In this study, immunoprecipitation was used to prepare two IgE samples from less than 1 mL of sera/plasma: one was from a patient with *PGM3* mutation, the other was from a patient with atopic dermatitis as a control subject. Then we used glycoproteomic strategies to study the glycans at each potential glycosylation site of the two samples. The results showed there are no significant differences between the two IgE samples. Moreover, our data combined with a recent study [14] show that IgE glycoproteomic spectra are similar among healthy controls, patients with allergy and the patient with HIES caused by *PGM3* mutation. These observations imply that, despite alterations occurring in the N-glycome of immune cells from patients with *PGM3* mutations, the elevated IgE in allergy and HIES may not be related to glycosylation on the antibody itself.

## Materials and methods

### Serum/plasma samples

Serum/plasma samples were collected from the Centre of Chronic Immunodeficiency (CCI), University Medical Center Freiburg, under human subject protocols approved by local ethics committees at University College London, the University of Freiburg and the Pasteur Institute of Tunis.

### IgE enrichment

IgE was enriched by immunoprecipitation using Pierce® Direct IP Kit (Thermo Scientific, Basingstoke, UK) according to the manufacturer’s instructions with some modifications. In order to immobilise an anti-IgE antibody on beads, the Pierce Spin Column was loaded with 30 μL AminoLink Plus Coupling resin and the liquid was removed by centrifuging at 1000 g for 1 min. Then, 300 μL 1× Coupling Buffer was used to wash the resin twice. After that, 185 μL H_2_O, 15 μL 20× Coupling Buffer, 100 μL IgE (4F4): sc-51994, mouse monoclonal antibody raised against IgE of human origin (Santa Cruz Biotechnology, Heidelberg, Germany) and 4.5 μL sodium cyanoborohydride were added onto the resin in the column and incubated at room temperature for 90 min in a rotator. The liquid was removed from the spin column by centrifuging at 1000 g for 1 min. The resin was washed twice with 300 μL 1× Coupling Buffer and 300 μL of 1× Quenching Buffer. Then, 300 μL of 1× Quenching Buffer and 4.5 μL of sodium cyanoborohydride were added and incubated at room temperature for 15 min on a rotator. The liquid was removed from the column again by centrifuging. Finally, the resin was washed once with 300 μL of 1× Coupling Buffer and 6 times with 200 μL washing solution. When the immobilization was completed, the resin was mixed with 600 μL of serum/plasma and gently rotated at 4 °C overnight. After that, 75 μL elution buffer was added to the resin and incubated for 10 min at room temperature. IgE was collected by centrifuging at 1000 g for 1 min.

### SDS-PAGE

The eluate was lyophilized and analysed by Novex® NuPAGE® SDS-PAGE Gel System (Invitrogen Ltd, Paisley, UK). Samples were dissolved in NuPAGE® LDS Sample Buffer, incubated at 70 °C for 10 min, loaded to Novex® NuPAGE® 3–8 % Tris-Acetate Mini Gels, and run at 150 V constant in Tris-Acetate SDS Running buffer. Gels were stained using Novex® Colloidal Blue Staining Kit. Gel bands of interest were chopped into 1 × 1 mm pieces, which were destained at room temperature using 50 mM ammonium hydrogen carbonate (Sigma-Aldrich, Poole, UK), pH 8.4 for 5 min, and mixed with equal amount of acetonitrile (Romil, Cambridge, UK) for another 5 min. The supernatant was discarded. The gel pieces were completely destained by repeating the two steps several times and were dried on a Thermo Savant SPD121P Speed Vac (Thermo Scientific, Basingstoke, UK). The samples in the gel pieces were reduced using 10 mM dithiothreitol (Roche Applied Science, East Sussex, UK) in ammonium hydrogen carbonate, pH 8.4 at 56 °C for 30 min. The dithiothreitol was then removed and the gel was washed and shrunk using 200 μL acetonitrile, and dried in the Speed Vac. Reduced samples were carboxymethylated by adding 200 μL 55 mM iodoacetic acid (Sigma-Aldrich, Poole, UK) in 50 mM ammonium hydrogen carbonate pH 8.4, and incubating in the dark for 30 min. After that, the iodoacetic acid solution was removed. The gel pieces were shrunk using 200 μL acetonitrile, and dried in the Speed Vac.

### In-gel digestion and (glyco)peptides extraction

The samples in the gel pieces were digested by trypsin (Promega, Hampshire, UK) or chymotrypsin (Promega, Hampshire, UK) into (glyco)peptides. Different proteolytic enzymes were utilized to facilitate analysis of individual glycosylation sites in glycopeptides. For trypsin digestion, 1/50 (*w*/*w*) of trypsin in 50 mM ammonium hydrogen carbonate, pH 8.4 was added to the dried gel pieces and incubated for 37 °C overnight. For chymotrypsin digestion 1/20 (*w*/*w*) of chymotrypsin in 50 mM ammonium hydrogen carbonate, pH 7.8 was added and incubated at 37 °C overnight. After the digestion, the supernatant was transferred to a new tube. Then 50 μL of 0.1 % (*v*/*v*) trifluoroacetic acid (Romil, Cambridge, UK) was added to the gel pieces and incubated at 37 °C for 10 min, followed by 100 μL acetonitrile and incubated at 37 °C for 15 min. The supernatant was pooled with the previous supernatant. This step was repeated twice. The combined supernatant was reduced to 10 μL in a Speed Vac for mass spectrometric analysis.

### Mass spectrometric analysis

MALDI-TOF/TOF was used for protein identification of the gel bands. The (glyco)peptides were mixed with α-CHCA (Sigma-Aldrich, Poole, UK) matrix (10 mg/mL in 50 % acetonitrile, 0.05 % trifluoroacetic acid), spotted on a MALDI plate, dried, and analysed using a 4800 MALDI-TOF/TOF™ Analyzer (Applied Biosystems, Darmstadt, Germany). The data were analysed using GPS Explorer™ Software (Applied Biosystems, Darmstadt, Germany). Both MS and MS/MS data were used to search 283,454 entries in release 54.2 of the SwissProt database with version 2.2 of the Mascot database search algorithm (www.matrixscience.com) with the following parameters: peptide masses were fixed as monoisotopic, partial oxidation of methionine residues was considered, partial carboxymethylation of cysteine residues was considered, the mass tolerance was set at 75 ppm for precursor ions and 0.1 Da for fragment ions and tryptic digests were assumed to have no more than one missed cleavage. Peptide matches from both MS and MS/MS data were used to generate probability-based Mowse protein scores. Scores of >55 were considered significant (*P* < 0.05). Glycoproteomic analysis was carried out according to a previous report [24], The (glyco) peptides were separated on a Pepmap C18 nanocapillary column (15 cm length, 75 mm internal diameter) fitted to a nano-HPLC system (LC Packings) connected to an ABI QSTAR Pulsar Hybrid LC/MS/MS system (Applied Biosystems/MDS Sciex). A gradient from 0.05 % (*v*/*v*) formic acid in 95.5 % (*v*/*v*) water/acetonitrile to 0.05 % (*v*/*v*) formic acid in 95.5 % (*v*/*v*) acetonitrile/water solution were employed. Data were analysed using Analyst QS Software.

## Results

### Preparation and proteomic identification of IgE

**Fig. 1:**
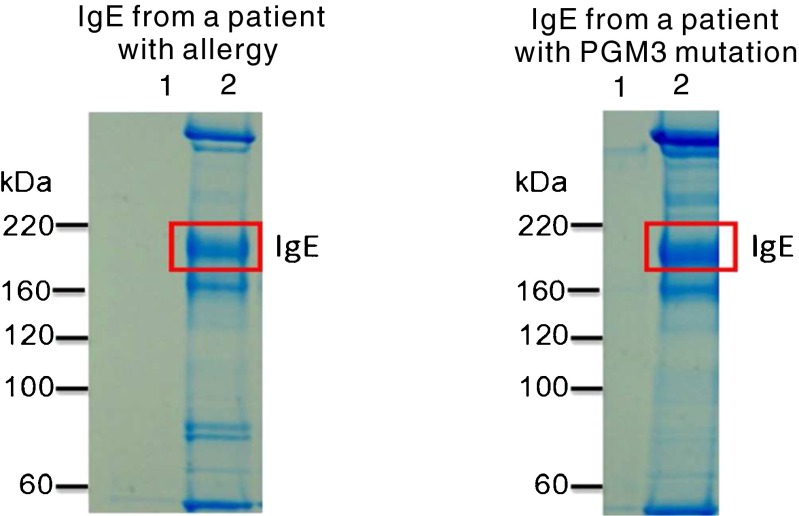
SDS-PAGE of immunoprecipitated IgE. 600 ul of serum from a patient with atopic dermatitis as a control subject and 600 ul of plasma from a patient with hyper IgE syndrome were used to prepare IgE. Elutions of immunoprecipitation were analyzed by non-reduced SDS-PAGE in 3–8 % Tris-acetate gel with Tris-acetate running buffer. *Lane 1*: Control resin. *Lane 2*: Immunoprecipitated IgE. A band was observed between 220 and 160 kDa in *Lane 2*, which is consistent with the molecular weight of IgE

IgE from a patient with *PGM3* mutation and a patient with atopic dermatitis as a control subject was enriched by immunoprecipitation and analysed by non-reduced SDS-PAGE, which is shown in Fig. 1. There was a clear band with molecular weight between 160 and 220 kDa in each sample, which is close to the predicted molecular weight of IgE (190 kDa). Each band was digested by trypsin and identified as human IgE by proteomic MALDI-TOF/TOF analysis (Supplementary Table 1 and Supplementary Table 2). As detailed below, the two IgE bands were subjected to glycoproteomic analysis by on-line nano-LC-MS. In addition Supplementary Table 8–11 compile the non-glycosylated IgE peptides also characterised in this analysis.

### Glycoproteomic analysis of Asn21

**Fig. 2:**
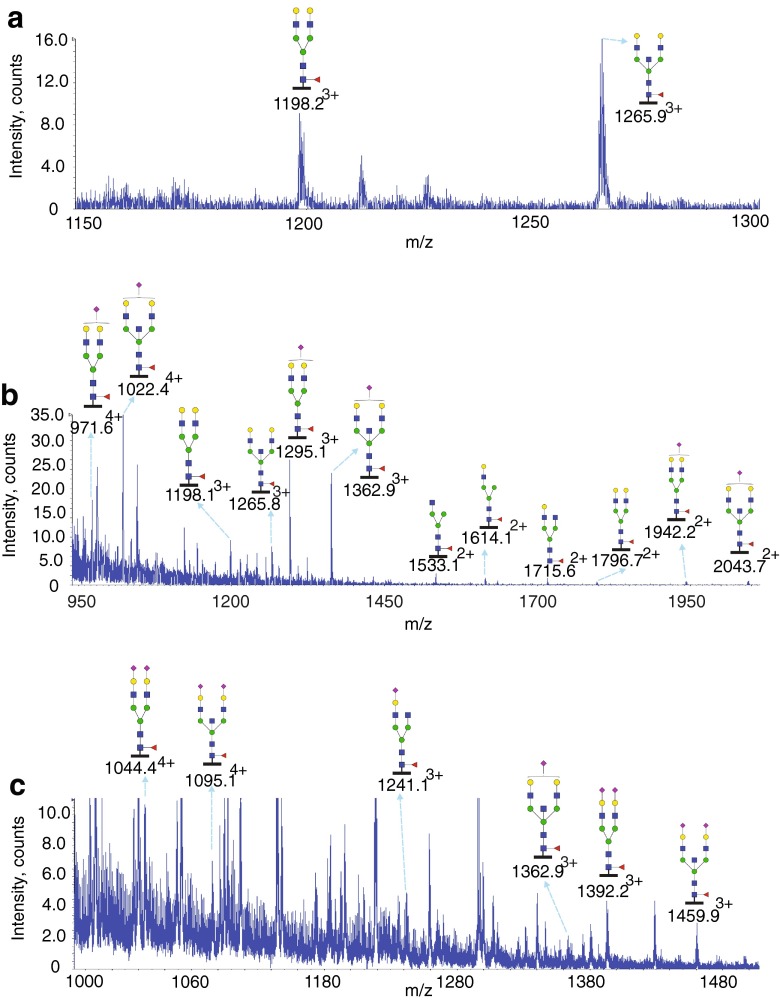
Glycosylation of IgE Asn21 from a *PGM3* patient. Data were acquired by LC/MS. The glycopeptides were produced by chymotrypsin digestion. The peptide backbone is presented as a *black bar* under each glycan (^12^TRCCKNIPS**N**ATSVTL^27^). The ions are in the form of M+nH^n+^. Glycopeptides were detected eluting at three time points: from 30.5 to 31.5 min (**a**), from 32.5 to 33.5 min (**b**) and from 35 to 37 min (**c**). The glycan structures were deduced according to the molecular weight, fragmention of glycopeptides by MS/MS analysis, previous glycomic analysis of released IgE N-glycans and knowledge of N-glycosylation biosynthetic pathways

Glycosylation at Asn21 of the IgE from a patient with *PGM3* mutation is shown in Fig. 2. Glycopeptides generated by chymotryptic digestion (^12^TRCCKNIPS**N**ATSVTL^27^) were detected eluting at three time points. Glycopeptides with non-sialylated glycans (Hex_5_HexNAc_4-5_Fuc) eluted between 30.5 and 31.5 min, those with mostly mono-sialylated glycans (NeuAc_0-1_Hex_5_HexNAc_4-5_Fuc) eluted between from 32.5 to 33.5 min, and those with mostly bi-sialylated glycans (NeuAc_1-2_Hex_4-5_HexNAc_4-5_Fuc) eluted between 35 and 37 min. All glycans were fucosylated and compositions consistent with bi-antennary structures dominated the spectra. The glycopeptides of the IgE from a control subject revealed a similar glycosylation profile (Table 1, Supplementary Figure 1, Supplementary Table 3).

**Table 1:**
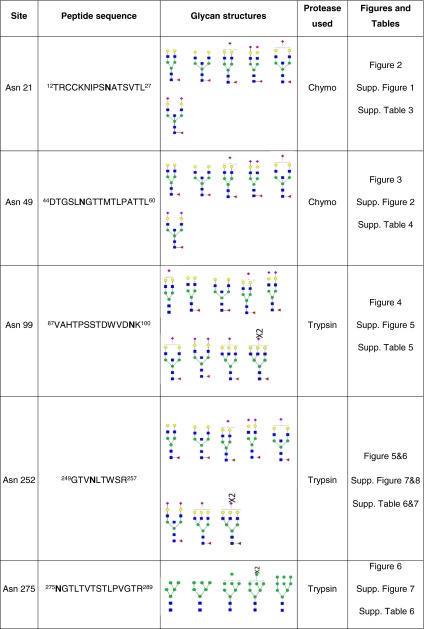
Summary of distribution of glycan structures among the IgE glycosylation sites mapped using our glycoproteomic strategy. For detailed description see Supplementary Tables 3–7

### Glycoproteomic analysis of Asn49

**Fig. 3:**
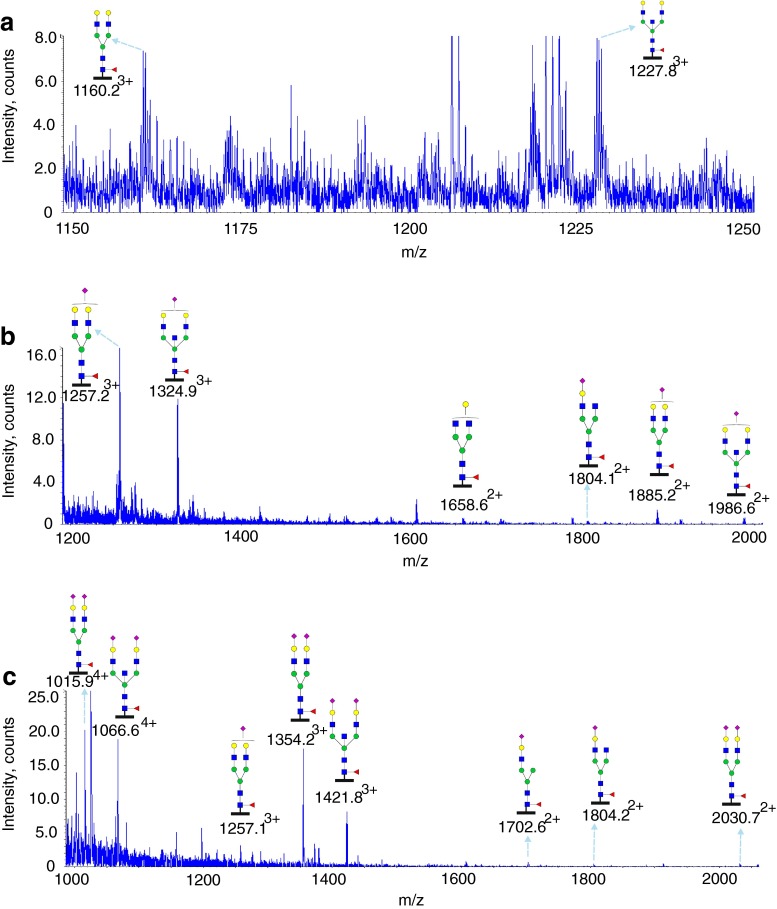
Glycosylation of IgE Asn49 from a *PGM3* patient. Data were acquired by LC/MS. The glycopeptides were produced by chymotrypsin digestion. The peptide backbone (^44^DTGSL**N**GTTMTLPATTL^60^) is presented as a *black bar* under each glycan. The ions are in the form of M+nH^n+^. Glycopeptides were detected eluting at three time points: from 37 to 38 min (**a**), from 40 to 42 min (**b**) and from 46 to 47.5 min (**c**). The glycan structures were deduced according to the molecular weight, fragmention of glycopeptides by MS/MS analysis, previous glycomic analysis of released IgE N-glycans and knowledge of N-glycosylation biosynthetic pathways

Glycosylation at Asn49 of the IgE from a patient with *PGM3* mutation is shown in Fig. 3 (Table 1). Glycopeptides generated by chymotryptic digestion (^44^DTGSL**N**GTTMTLPATTL^60^) were detected eluting at three time points. Glycopeptides with non-sialylated glycans (Hex_5_HexNAc_4-5_Fuc) eluted between 37 and 38 min, those with mostly mono-sialylated glycans (NeuAc_0-1_Hex_4-5_HexNAc_4-5_Fuc) eluted between from 40 to 42 min, and those with mostly bi-sialylated glycans (NeuAc_1-2_Hex_4-5_HexNAc_4-5_Fuc) eluted between 46 and 47.5 min. All glycans were fucosylated and compositions consistent with bi-antennary structures dominated the spectra. Again the glycopeptides of the IgE from a control subject revealed a similar glycosylation profile (Table 1, Supplementary Figure 2, Supplementary Table 4). The N-glycosylation profiles of both IgE samples are very similar at Asn22 and Asn49 with non-sialylated, mono-sialylated and bi-sialylated glycopeptides.

To provide additional levels of structural definition MS/MS analysis was performed on selected molecular ions. Exemplary ESI-CID-MS/MS data of *m*/*z* 1066.6^4+^ are shown in Supplementary Figure 3. The spectrum is dominated by glycan fragment ions (*m*/*z* 168, 186, 204 derived from GlcNAc; *m*/*z* 256, 274 and 292 derived from sialic acid and *m*/*z* 366 derived from LacNAc). Moreover, two C-terminal peptide fragments were detected at *m*/*z* 502 and 615. Collectively the data show that *m*/*z* 1066.6^4+^ is the chymotryptic glycopeptide spanning Asn-49 carrying a core fucosylated, bisected, disialylated biantennary glycan.

### Glycoproteomic analysis of Asn99

**Fig. 4:**
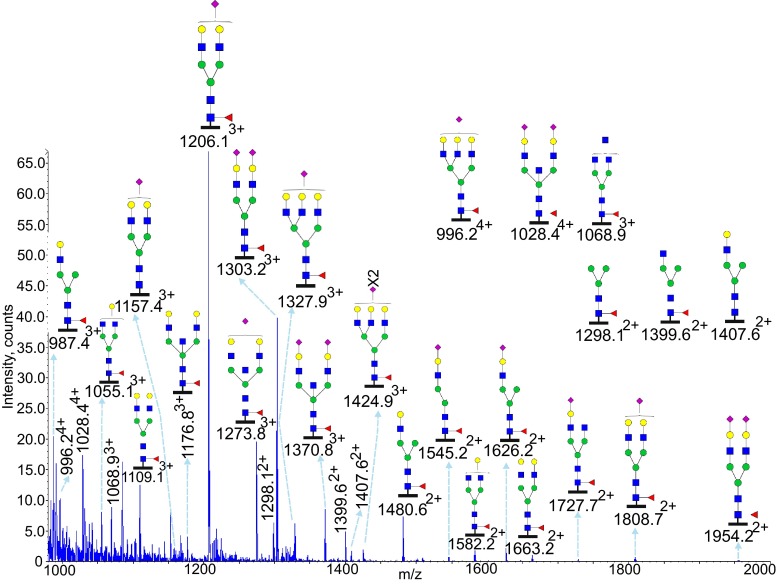
Glycosylation of IgE Asn99 from a *PGM3* patient. Data were acquired by LC/MS. The glycopeptides were produced by trypsin digestion. The peptide backbone (^87^VAHTPSSTDWVD**N**K^100^) is presented as a *black bar* under each glycan. The ions are in the form of M+nH^n+^. Glycopeptides were detected eluting between 21 and 23 min. The glycan structures were deduced according to the molecular weight, fragmention of glycopeptides by MS/MS analysis, previous glycomic analysis of released IgE N-glycans and knowledge of N-glycosylation biosynthetic pathways

A greater diversity of N-glycan structures was observed at Asn99 compared to Asn 21 and Asn49 in the two IgE samples. As indicated in Fig. 4 (Table 1 and Supplementary Table 5) glycopeptides generated by tryptic digestion (^87^VAHTPSSTDWVD**N**K^100^) of IgE from a patient with *PGM3* mutation were detected eluting between 21 to 23 min. Compositionally the major N-glycans had bi-antennary structures with fucose and sialic acid, for example the two most abundant molecular ions at *m*/*z* 1206.1^3+^ and 1303.2^3+^. A non-fucosylated glycan at *m*/*z* 1157.4^3+^ was detected in both samples with low intensity. A small amount of non-sialylated glycans were detected, such as the peaks at *m*/*z* 1109.1^3+^ and *m*/*z* 1176.8^2+^. Potential tri-antennary glycans were observed at *m*/*z* 996.2^4+^, *m*/*z* 1327.9^3+^, and 1424.9^3+^ as well as compositions consistent with mono-antennary complex structures such as *m*/*z* 1480.6^2+^, *m*/*z* 1545.2^2+^, and 1626.2^2+^. ESI-CID-MS/MS of the glycopeptide at *m*/*z* 1206.1^3+^ is shown in Supplementary Figure 4. Similar to the fragment patterns observed in Supplementary Figure 3, glycan fragments at low *m*/*z* range dominated the spectra. The peptide containing a GlcNAc residue (*m*/*z* 880.4^2+^ and *m*/*z* 1759.9^+^) and the peptide with a GlcNAc and a Fuc residue (*m*/*z* 1905.8^+^) provide strong evidence for the presence of core fucosylation. In addition, peptide y ions with glycan fragments were detected (*m*/*z* 982.1^2+^, 1063.0^2+^, 1144.0^2+^ and *m*/*z* 1351.6^+^), which provides further information on both peptide sequence and glycan core structures. Again the glycopeptides of the IgE from a control subject revealed a similar glycosylation profile (Table 1, Supplementary Figure 5).

### Glycoproteomic analysis of Asn252 and Asn275

**Fig. 5:**
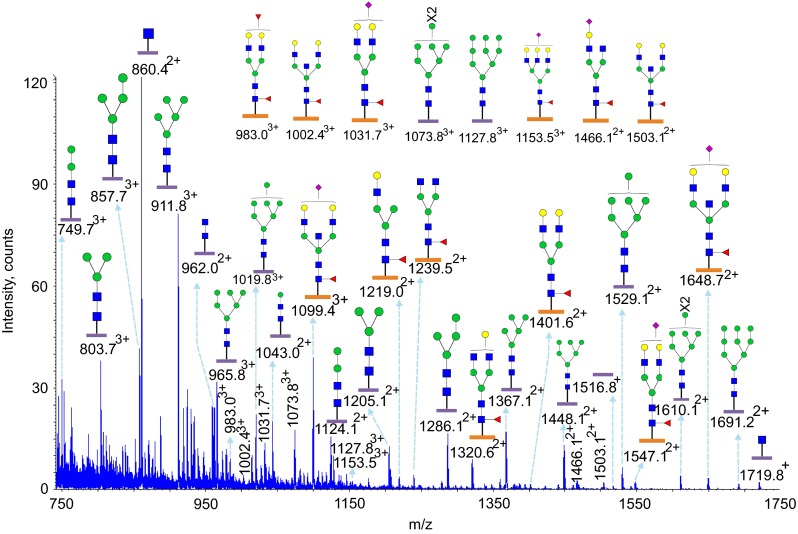
Glycosylation of IgE Asn252 and Asn275 from a *PGM3* patient. Data were acquired by LC/MS. The glycopeptides were produced by trypsin digestion. Peptide backbones with Asn252 (^249^GTV**N**LTWSR^257^) and Asn275 (^275^ **N**GTLTVTSTLPVGTR^289^) are presented as an *orange bar* and a *purple bar* respectively under each glycan. The ions are in the form of M+nH^n+^. Glycopeptides were eluted between 27.5 and 32 min. The glycan structures were deduced according to the molecular weight, fragmention of glycopeptides by MS/MS analysis, previous glycomic analysis of released IgE N-glycans and knowledge of N-glycosylation biosynthetic pathways

**Fig. 6:**
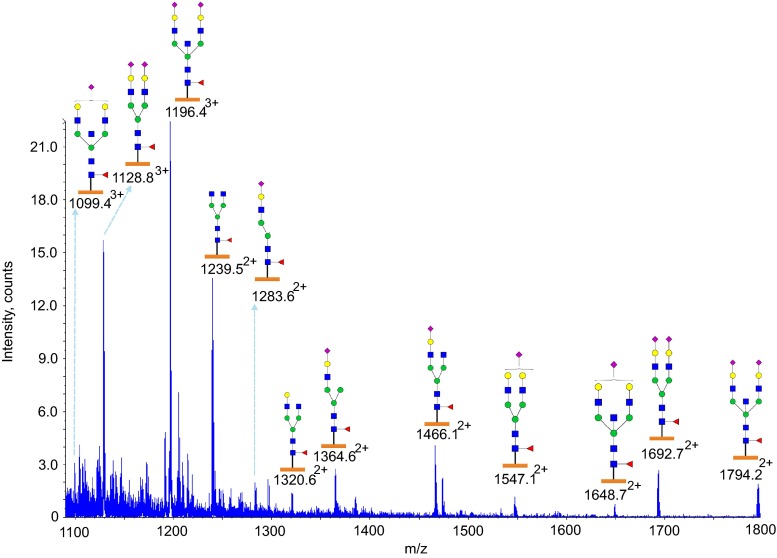
Glycosylation of IgE Asn252 from a *PGM3* patient. Data were acquired by LC/MS. The glycopeptides were produced by trypsin digestion. The peptide backbone with (^249^GTV**N**LTWSR^257^) is presented as an *orange bar* under each glycan. The ions are in the form of M+nH^n+^. The glycopeptides were eluted from 34.5 to 36.5 min. The glycan structures were deduced according to the molecular weight, fragmention of glycopeptides by MS/MS analysis, previous glycomic analysis of released IgE N-glycans and knowledge of N-glycosylation biosynthetic pathways

Glycosylation at Asn252 of the IgE from a patient with *PGM3* mutation is shown in Figs. 5 and 6 (Table 1, Supplementary Table 6, 7). Glycopeptides generated by tryptic digestion (^249^GTV**N**LTWSR^257^) were detected eluting at two time points. At the first time point between 27.5 and 32 min glycopeptides containing Asn275 (^275^**N**GTLTVTSTLPVGTR^289^) were also observed. Glycosylation at Asn252 (NeuAc_0-1_Hex_4-6_HexNAc_4-5_Fuc_1_) contained mainly mono-sialylated bi-antennary glycans, such as *m*/*z* 1099.4^3+^ and *m*/*z* 1031.7^3+^, except for a tri-antennary glycan with a single sialic acid at *m*/*z* 1153.5^3+^. ESI-CID-MS/MS of *m*/*z* 1002.4^+^ of the PGM3 patient (Supplementary Figure 6) confirmed the core fucosylated, non-sialylated glycan structure as annotated in the MS studies (Fig. 5). The second time point between 34.5 min and 36.5 min, contained exclusively Asn252 containing glycopeptides. Mainly bi-sialyated glycans were observed (NeuAc_0-2_Hex_3-5_HexNAc_3-5_Fuc_1_), as well as compositions consistent with mono-antennary complex structures such as *m*/*z* 1364.6^2+^.

In contrast N-glycans at Asn275 were high mannose glycans (Hex_5-9_HexNAc_2_) and paucimannose glycans (Hex_2-4_HexNAc_2_) (Fig. 6, Table 1, Supplementary Table 7). Glycopeptides of the IgE from a control subject revealed a similar glycosylation profile (Table 1, Supplementary Figure 7, 8).

### Analysis of Asn264

Glycosylation at Asn264 was not observed and the unglycosylated tryptic peptide (^258^ASGKPV**N**HSTR^268^), was detected in both IgE samples (Supplementary Figure 9, Supplementary Figure 10) at *m*/*z* 385.2^3+^ and *m*/*z* 577.3^2+^. The MS/MS of *m*/*z* 385.2^3+^ confirmed the peptide sequence (Supplementary figure 11).

## Discussion

We previously demonstrated that the overall IgE N-glycan profiles were similar between a patient with *PGM3* mutation and patient with atopic dermatitis as a control subject [23]. In this paper we have now shown by glycoproteomic studies that the two types of IgE had similar site specific N-glycosylation. These observations together with a recent report [14] demonstrate that IgE glycosylation is similar among healthy controls, allergy patients and patients with *PGM3* related hyper IgE syndrome. Our glycoproteomic studies found that Asn264 was not occupied by N-glycans and Asn275 had only high mannose glycans, which is consistent with all previous site specific glycosylation studies [10–12, 14]. A trace amount of Fuc was detected in an myeloma IgE at Asn99 reported in 1970s [10–12], which is in contrast to the latest glycoproteomic report [14]. Our studies again found the glycans were mostly core fucosylated at this site. A HPLC based glycomic analysis [13] of an allergic IgE indicated that all the seven potential N-glycosylation sites were glycosylated, while our results together with previous studies [10–12] and the most recent report [14] showed Asn 264 is not glycosylated. Asn146 was not mapped in this study, which coincides with a previous report that this site cannot be mapped by trypsin or chymotrypsin digestion [14]. Nevertheless, despite not characterising glycosylation at Asn146 we still conclude that IgE glycosylation does not change in allergy and *PGM3* related hyper IgE syndrome, because we have both the overall glycan profile of IgE and glycoproteomic data from 6 of the 7 glycosylation sites in the glycoproteomic data. Combining the two sets of data, we feel it is highly unlikely that the glycosylation at Asn146 does change.

Both glycomic [23] and glycoproteomic studies showed *PGM3* mutation did not affect IgE glycosylation, although elevated IgE is a key symptom in these patients. A possible explanation is that the major complex N-glyans on IgE have bi-antennary structures. Our previous studies of B cells and neutrophils from *PGM3* mutation patients have shown that tri-/tetra-antennary glycans are most sensitive to biochemical changes caused by *PGM3* mutation, while bi-antennary glycans are not as sensitive [23]. The elevated IgE may be caused by decreased tri-/tetra-antennary glycans on other glycoproteins that are involved in IgE production. In addition, it has been known that O-GlcNAcylation is hypersensitive to UDP-GlcNAc concentration [25]. A mouse model with hypomorphic mutations in *Pgm3* has shown decreased O-GlcNAcylation [26]. Therefore, impairment of O-GlcNAcylation could be another cause of the elevated IgE in the patients.

## Electronic supplementary material

Below is the link to the electronic supplementary material.

ESM 1 (DOCX 1460 kb)
