# Supplementary material for: Glycoproteomic studies of IgE from a novel hyper IgE syndrome linked to PGM3 mutation
Source: Glycoconj J. 2015 Dec 19;33:447–56. doi: 10.1007/s10719-015-9638-y (PMC4891365; doi:10.1007/s10719-015-9638-y)
Supplement: Supplementary file 1 — (DOCX 1460 kb) [file 10719_2015_9638_MOESM1_ESM.docx]

**Glycoproteomic studies of IgE from a novel hyper IgE syndrome linked to PGM3 mutation**

Gang Wu^1,2^, Paul G. Hitchen^1^, Maria Panico^1^, Simon J. North^1^, Mohamed-Ridha Barbouche^3^, Daniel Binet^4^, Howard R. Morris^1,4^, Anne Dell^1^, and Stuart M. Haslam^1^*

From the ^1^Department of Life Sciences, Imperial College London, South Kensington Campus, London SW7 2AZ, United Kingdom, ^2^Division of Cell Signalling and Immunology, School of Life Sciences, University of Dundee, Dundee DD1 5EH, UK, Laboratory of Immunopathology, Vaccinology and Molecular Genetics, Pasteur Institute of Tunis and University Tunis El Manar, Tunis, Tunisia, ^4^MS-RTC (Mass Spectrometry Research and Training Centre), Suite 3.1 Lido Medical Centre, St. Saviours Road, Jersey, JE2 7LA, United Kingdom

*To whom correspondence should be addressed: Department of Life Sciences, Faculty of Natural Sciences, Imperial College London, South Kensington Campus, London, SW7 2AZ, United Kingdom. Tel.: +4420-75945222; Fax: +4420-72250458; E-mail: [s.haslam@imperial.ac.uk](mailto:s.haslam@imperial.ac.uk).

Glycoconjugate Journal


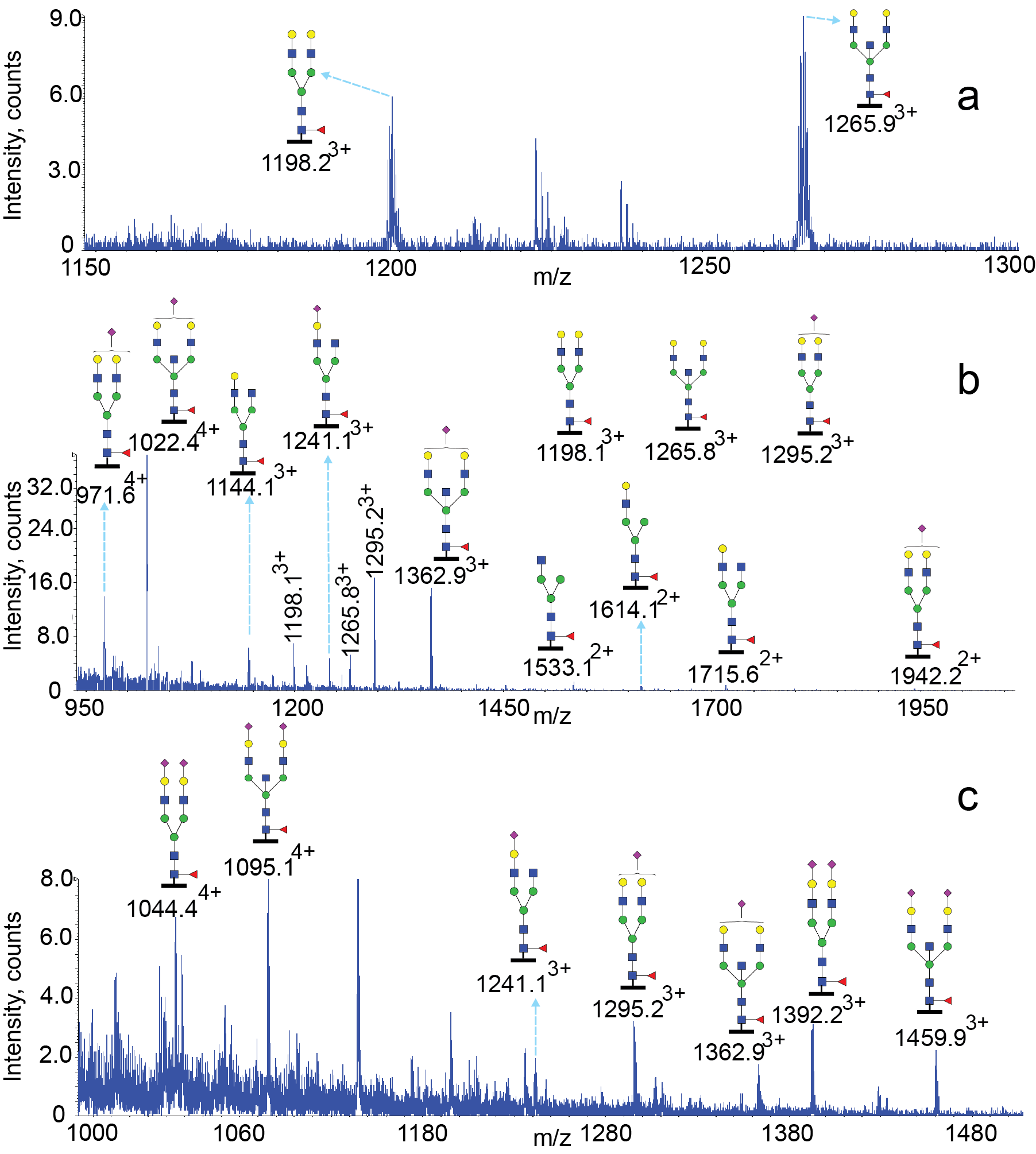


**Supplementary Fig. 1** Glycosylation of the IgE at Asn21 from a control subject

Data were acquired by LC/MS. The glycopeptides were produced by chymotrypsin digestion. Peptide backbone is presented as a black bar under each glycan (^12^TRCCKNIPS**N**ATSVTL^27^). The ions are in the form of M+nH^n+^. Glycopeptides were detected eluting at three time points: a from 37.5 min to 38.5 min, b from 40 min to 41 min and c was from 43 min to 45 min. The glycan structures were deduced according to the molecular weight, fragmention of glycopeptides by MS/MS analysis, previous glycomic analysis of released IgE N-glycans and knowledge of N-glycosylation biosynthetic pathways


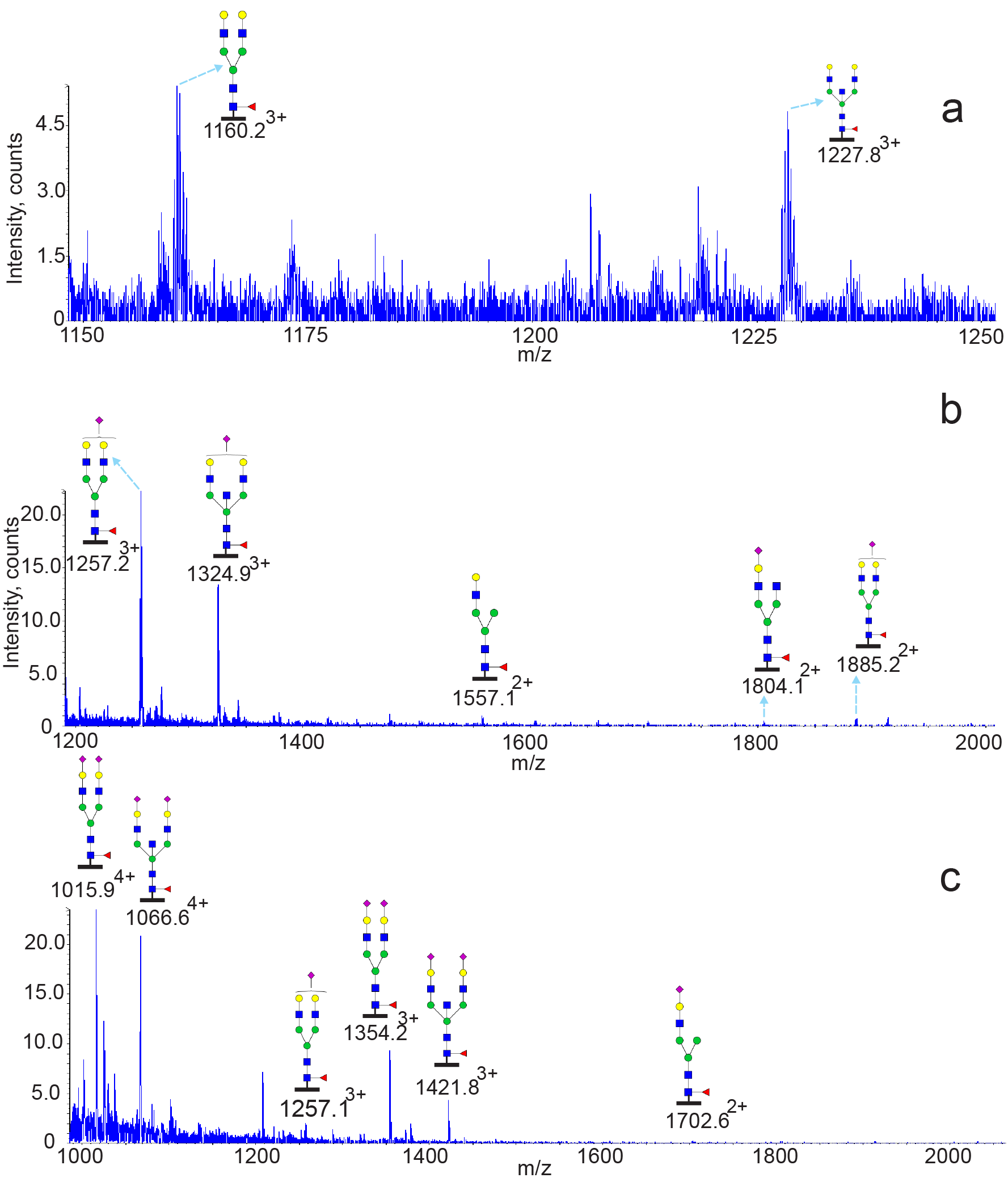


**Supplementary Fig. 2** Glycosylation of the IgE at Asn49 from a control subject.

Data were acquired by LC/MS. The glycopeptides were produced by chymotrypsin digestion. Peptide backbone (^44^DTGSL**N**GTTMTLPATTL^60^) is presented as a black bar under each glycan. The ions are in the form of M+nH^n+^. Glycopeptides were detected eluting at three time points: a from 47.2 min to 48.2 min, b from 51.4 min to 52.4 min and c was from 57 min to 59 min. The glycan structures were deduced according to the molecular weight, fragmention of glycopeptides by MS/MS analysis, previous glycomic analysis of released IgE N-glycans and knowledge of N-glycosylation biosynthetic pathways

**Supplementary Fig. 3** ESI-CID-MS/MS of the glycopeptide molecular ion at m/z 1066.6^4+^ derived from IgE Asn49 from a *PGM3* patient

The annotated ions are all 5. The spectrum is dominated by glycan fragments, such as GlcNAc at m/z 204.1^+^, sialic acid at m/z 274.1^+^ and LacNAc unit at m/z 366.1^+^. Losses of water molecules from GlcNAc and sialic acid were detected.

**Supplementary Fig. 4**. ESI-CID-MS/MS the glycopeptide at m/z 1206.1^3+^ derived from IgE Asn99 from a *PGM3* patient

The charge state of annotated ions are indicated. The major peaks are glycan fragments at low m/z range, such as the peaks at m/z 204.1^+^ (GlcNAc), m/z 292.1^+^ (sialic acid) and m/z 366.1^+^ (LacNAc unit). Losses of water molecules from the GlcNAc and sialic acid were observed. Peptide with glycan fragments were detected at m/z 880.4^2+^ (peptide+GlcNAc+2H^+^), m/z 1759.9^+^ (peptide+GlcNAc+H^+^) and m/z 1905.8^+^ (peptide+GlcNAc+Fuc+H^+^). Fragmentation on both peptide backbone and glycans were detected at m/z 982.1^2+^, 1063.0^2+^, 1144.0^2+^ and m/z 1351.6^+^. Peptide backbone (^87^VAHTPSSTDWVD**N**K^100^) is indicated as a black bar under each glycan.


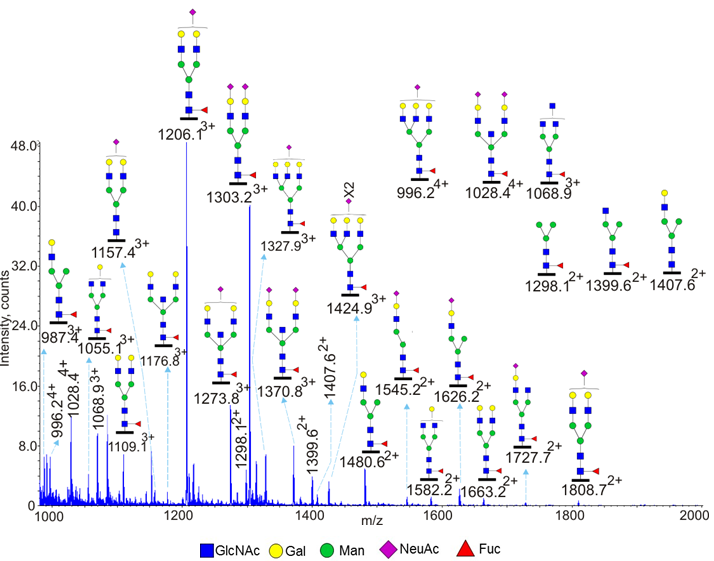


**Supplementary Fig. 5** Glycosylation at Asn99 of the IgE from a control subject

Data were acquired by LC/MS. The glycopeptides were produced by trypsin digestion. The peptide backbone (^87^VAHTPSSTDWVD**N**K^100^) is presented as a black bar under each glycan. The ions are in the form of M+nH^n+^. The glycopeptides were eluted from 24 min to 26 min. The glycan structures were deduced according to the molecular weight, fragmention of glycopeptides by MS/MS analysis, previous glycomic analysis of released IgE N-glycans and knowledge of N-glycosylation biosynthetic pathways
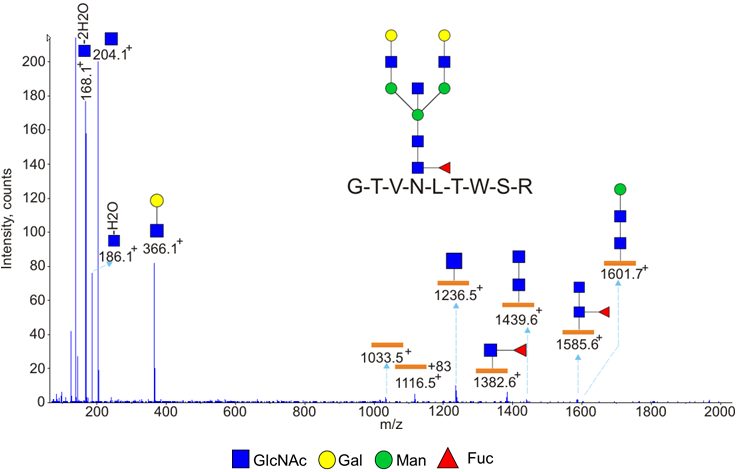


**Supplementary Fig. 6** ESI-CID-MS/MS of an IgE glycopeptide at m/z 1002.4^3+^ from *PGM3* patient

The annotated ions are all singly charged. The spectrum is dominated by glycan fragments, such as GlcNAc at m/z 204 and LacNAc unit at m/z 366. Peptide backbone was detected at 1033.5^+^. Peptide + GlcNAc + H^+^, peptide + GlcNAcFuc + H^+^, peptide + GlcNAc_2_ + H^+^, peptide + GlcNAc_2_Fuc + H^+^, and peptide+GlcNAc_2_+Man+H^+^ were observed.


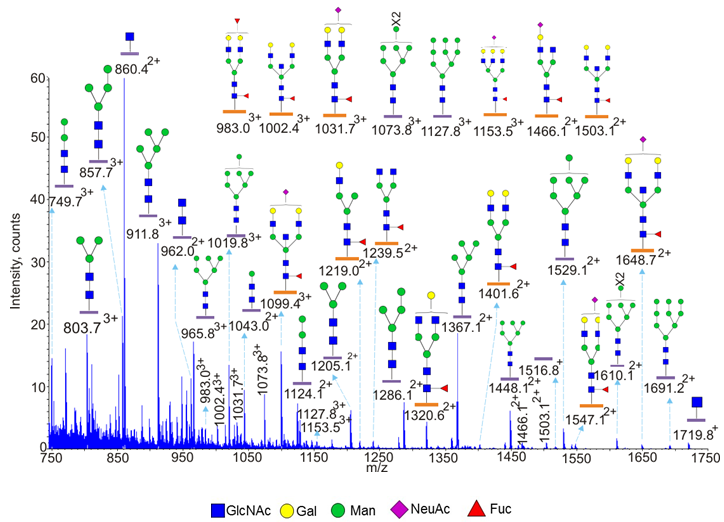


**Supplementary Fig. 7** Glycosylation at Asn252 and Asn275 of the IgE from a from a control subject

Data were acquired by LC/MS. The glycopeptides were produced by trypsin digestion. Peptide backbones with Asn252 (^249^GTV**N**LTWSR^257^) and Asn275 (^275^**N**GTLTVTSTLPVGTR^289^) are presented as an orange bar and a purple bar respectively under each glycan. The ions are in the form of M+nH^n+^. Glycopeptides were eluted between 29.5 min and 34 min. The glycan structures were deduced according to the molecular weight, fragmention of glycopeptides by MS/MS analysis, previous glycomic analysis of released IgE N-glycans and knowledge of N-glycosylation biosynthetic pathways


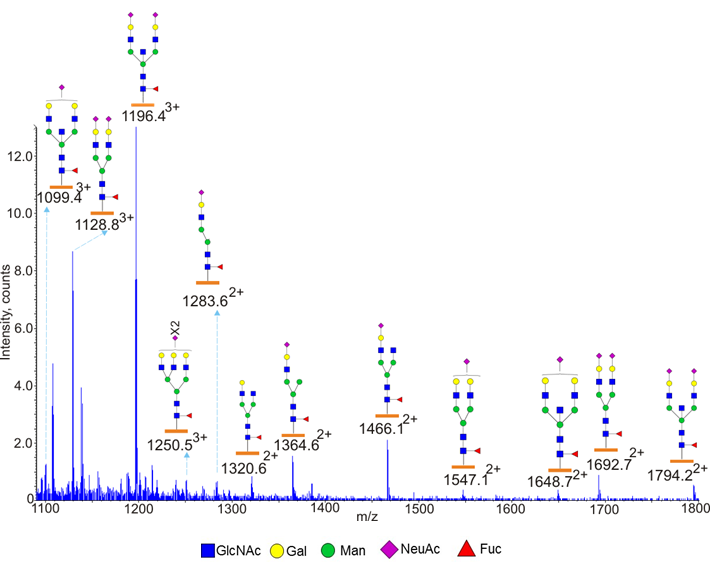


**Supplementary Fig. 8** Glycosylation at Asn252 (the second fraction) of the IgE from a patient with allergy

Data were acquired by LC/MS. The glycopeptides were produced by trypsin digestion. The peptide backbone with (^249^GTV**N**LTWSR^257^) is presented as an orange bar under each glycan. The ions are in the form of M+nH^n+^. The glycopeptides were eluted from 37 min to 39 min. Bi-sialylated structures were mainly observed. The glycan structures were deduced according to the molecular weight, fragmention of glycopeptides by MS/MS analysis, previous glycomic analysis of released IgE N-glycans and knowledge of N-glycosylation biosynthetic pathways


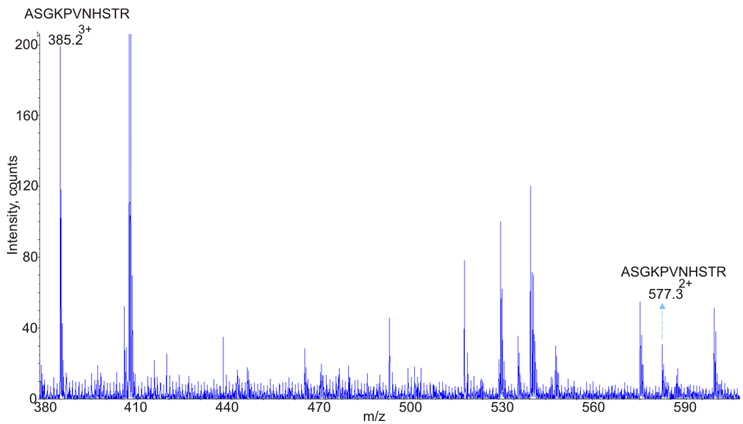


**Supplementary Fig. 9** Asn264 detected in an unglycosylated tryptic peptide (^258^ASGKPV**N**HSTR^268^), from the IgE of a *PGM3* patient. The peptide eluted from 36 min to 40 min. Data were acquired by LC/MS.


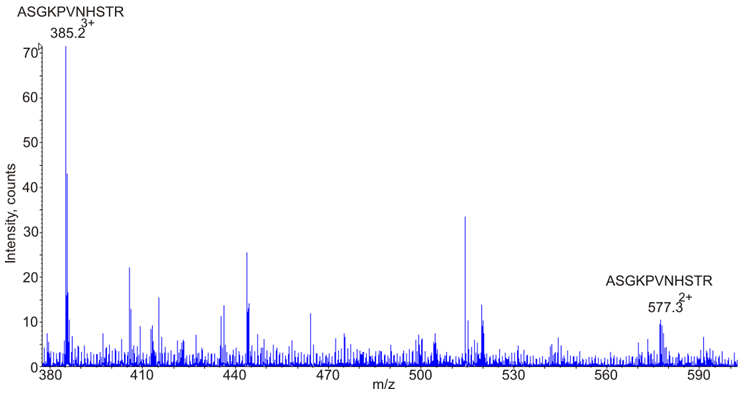


**Supplementary Fig. 10** Asn264 detected in an unglycosylated tryptic peptide (^258^ASGKPVNHSTR^268^), from the IgE of a control subject. The peptide was eluted from 38 min to 42 min. Data were acquired by LC/MS.


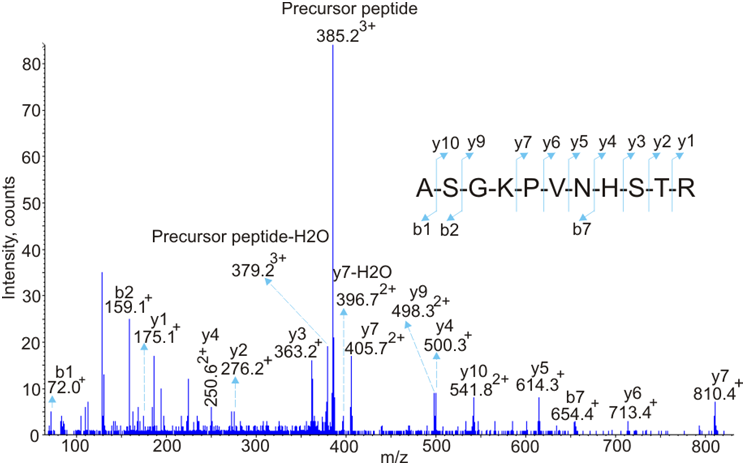


**Supplementary Fig. 11** MS/MS analysis of m/z 385.2^3+^ from the control subject IgE peptide. The masses of fragments provide strong evidence for the peptide sequence shown in the spectrum.

**Supplementary Table 1** Proteomic identification of IgE from control subject


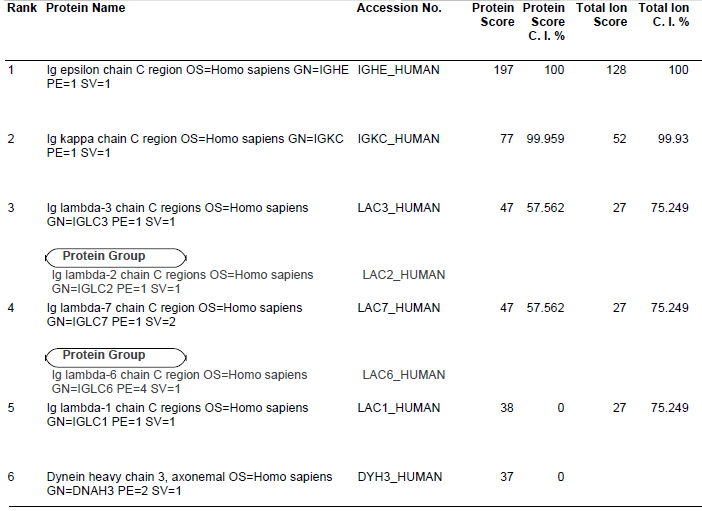


| **Supplementary Table 1a :** IGHE_HUMAN : Ig epsilon chain C region OS=Homo sapiens GN=IGHE PE=1 SV=1 | | | | | | | |
| --- | --- | --- | --- | --- | --- | --- | --- |
|  |  |  |  |  |  |  |  |
| Peptide Number | Calculated Mass | Observed Mass | Match Error Da | Start Sequence Position | End Sequence Position | Sequence | Modification |
| 1 | 813.4002 | 813.4205 | 0.0203 | 184 | 189 | HWLSDR |  |
| 2 | 859.3436 | 859.3627 | 0.0191 | 81 | 86 | QMFTCR | Oxidation (M)[2], Carboxymethyl (C)[5] |
| 3 | 956.5424 | 956.5679 | 0.0255 | 301 | 308 | VTHPHLPR |  |
| 4 | 1003.4995 | 1003.5288 | 0.0293 | 381 | 389 | GSGFFVFSR |  |
| 5 | 1003.4995 | 1003.5288 | 0.0293 | 381 | 389 | GSGFFVFSR |  |
| 6 | 1033.5425 | 1033.5688 | 0.0263 | 249 | 257 | GTVNLTWSR |  |
| 7 | 1457.6002 | 1457.6403 | 0.0401 | 290 | 300 | DWIEGETYQCR | Carboxymethyl (C)[10] |
| 8 | 1457.6002 | 1457.6403 | 0.0401 | 290 | 300 | DWIEGETYQCR | Carboxymethyl (C)[10] |
| 9 | 1480.7502 | 1480.7848 | 0.0346 | 407 | 420 | AVHEAASPSQTVQR |  |
| 10 | 1480.7502 | 1480.7848 | 0.0346 | 407 | 420 | AVHEAASPSQTVQR |  |
| 11 | 1611.7108 | 1611.7551 | 0.0443 | 395 | 406 | AEWEQKDEFICR | Carboxymethyl (C)[11] |
| 12 | 1611.7108 | 1611.7551 | 0.0443 | 395 | 406 | AEWEQKDEFICR | Carboxymethyl (C)[11] |
| 13 | 1848.8915 | 1848.9395 | 0.048 | 322 | 338 | AAPEVYAFATPEWPGSR |  |
| 14 | 1848.8915 | 1848.9395 | 0.048 | 322 | 338 | AAPEVYAFATPEWPGSR |  |
| 15 | 1925.028 | 1925.0831 | 0.0551 | 216 | 232 | GVSAYLSRPSPFDLFIR |  |
| 16 | 2166.9285 | 2167.0244 | 0.0959 | 190 | 207 | TYTCQVTYQGHTFEDSTK | Carboxymethyl (C)[4] |
| 17 | 2295.0234 | 2295.0981 | 0.0747 | 190 | 208 | TYTCQVTYQGHTFEDSTKK | Carboxymethyl (C)[4] |

| **Supplementary Table 1b** : IGKC_HUMAN : Ig kappa chain C region OS=Homo sapiens GN=IGKC PE=1 SV=1 | | | | | | | |
| --- | --- | --- | --- | --- | --- | --- | --- |
|  |  |  |  |  |  |  |  |
| Peptide Number | Calculated Mass | Observed Mass | Match Error Da | Start Sequence Position | End Sequence Position | Sequence | Modification |
| 1 | 1798.8793 | 1798.9276 | 0.0483 | 19 | 34 | SGTASVVCLLNNFYPR | Carboxymethyl (C)[8] |
| 2 | 1798.8793 | 1798.9276 | 0.0483 | 19 | 34 | SGTASVVCLLNNFYPR | Carboxymethyl (C)[8] |
| 3 | 1876.9109 | 1876.9607 | 0.0498 | 83 | 99 | VYACEVTHQGLSSPVTK | Carboxymethyl (C)[4] |
| 4 | 1946.027 | 1946.089 | 0.062 | 1 | 18 | TVAAPSVFIFPPSDEQLK |  |
| 5 | 1946.027 | 1946.089 | 0.062 | 1 | 18 | TVAAPSVFIFPPSDEQLK |  |

| **Supplementary Table 1c** : LAC3_HUMAN : Ig lambda-3 chain C regions OS=Homo sapiens GN=IGLC3 PE=1 SV=1 | | | | | | | |
| --- | --- | --- | --- | --- | --- | --- | --- |
|  |  |  |  |  |  |  |  |
| Peptide Number | Calculated Mass | Observed Mass | Match Error Da | Start Sequence Position | End Sequence Position | Sequence | Modification |
| 1 | 865.3607 | 865.3652 | 0.0045 | 99 | 106 | TVAPTECS | Carboxymethyl (C)[7] |
| 2 | 1743.8589 | 1743.9191 | 0.0602 | 66 | 80 | YAASSYLSLTPEQWK |  |
| 3 | 1743.8589 | 1743.9191 | 0.0602 | 66 | 80 | YAASSYLSLTPEQWK |  |
| 4 | 1986.0178 | 1986.0712 | 0.0534 | 5 | 23 | AAPSVTLFPPSSEELQANK |  |

| **Supplementary Table 1d** : LAC7_HUMAN : Ig lambda-7 chain C region OS=Homo sapiens GN=IGLC7 PE=1 SV=2 | | | | | | | |
| --- | --- | --- | --- | --- | --- | --- | --- |
|  |  |  |  |  |  |  |  |
| Peptide Number | Calculated Mass | Observed Mass | Match Error Da | Start Sequence Position | End Sequence Position | Sequence | Modification |
| 1 | 835.3502 | 835.3136 | -0.0366 | 99 | 106 | TVAPAECS | Carboxymethyl (C)[7] |
| 2 | 1743.8589 | 1743.9191 | 0.0602 | 66 | 80 | YAASSYLSLTPEQWK |  |
| 3 | 1743.8589 | 1743.9191 | 0.0602 | 66 | 80 | YAASSYLSLTPEQWK |  |
| 4 | 1986.0178 | 1986.0712 | 0.0534 | 5 | 23 | AAPSVTLFPPSSEELQANK |  |

| **Supplementary Table 1e** : LAC1_HUMAN : Ig lambda-1 chain C regions OS=Homo sapiens GN=IGLC1 PE=1 SV=1 | | | | | | | |
| --- | --- | --- | --- | --- | --- | --- | --- |
|  |  |  |  |  |  |  |  |
| Peptide Number | Calculated Mass | Observed Mass | Match Error Da | Start Sequence Position | End Sequence Position | Sequence | Modification |
| 1 | 865.3607 | 865.3652 | 0.0045 | 99 | 106 | TVAPTECS | Carboxymethyl (C)[7] |
| 2 | 1743.8589 | 1743.9191 | 0.0602 | 66 | 80 | YAASSYLSLTPEQWK |  |
| 3 | 1743.8589 | 1743.9191 | 0.0602 | 66 | 80 | YAASSYLSLTPEQWK |  |

| **Supplementary Table 1e** : DYH3_HUMAN : Dynein heavy chain 3, axonemal OS=Homo sapiens GN=DNAH3 PE=2 SV=1 | | | | | | | |
| --- | --- | --- | --- | --- | --- | --- | --- |
|  |  |  |  |  |  |  |  |
| Peptide Number | Calculated Mass | Observed Mass | Match Error Da | Start Sequence Position | End Sequence Position | Sequence | Modification |
| 1 | 803.3716 | 803.321 | -0.0506 | 599 | 605 | NQVGPCK | Carboxymethyl (C)[6] |
| 2 | 806.444 | 806.3671 | -0.0769 | 2246 | 2252 | MLVQATK | Oxidation (M)[1] |
| 3 | 849.4061 | 849.3672 | -0.0389 | 168 | 174 | NKEDSTR |  |
| 4 | 956.5775 | 956.5679 | -0.0096 | 1632 | 1640 | ALLDVNLAK |  |
| 5 | 1033.5411 | 1033.5688 | 0.0277 | 3268 | 3276 | VLSEEISEK |  |
| 6 | 1103.5765 | 1103.6265 | 0.05 | 4031 | 4040 | TMQIGESLPK |  |
| 7 | 1190.7031 | 1190.6583 | -0.0448 | 721 | 730 | ELVSLIEFLK |  |
| 8 | 1211.6321 | 1211.6801 | 0.048 | 325 | 334 | APVPWHSVYR |  |
| 9 | 1218.6147 | 1218.6237 | 0.009 | 780 | 789 | DQAEMDLIKR |  |
| 10 | 1319.6624 | 1319.6128 | -0.0496 | 2910 | 2921 | EAEGKLAAQMQK | Oxidation (M)[10] |
| 11 | 1457.6829 | 1457.6403 | -0.0426 | 1865 | 1877 | DSYMDTLPSSLTK |  |
| 12 | 1457.7781 | 1457.6403 | -0.1378 | 1133 | 1145 | ILVAADQPRMAEK | Oxidation (M)[10] |
| 13 | 1473.6777 | 1473.6422 | -0.0355 | 1865 | 1877 | DSYMDTLPSSLTK | Oxidation (M)[4] |
| 14 | 1480.7253 | 1480.7848 | 0.0595 | 404 | 415 | WIPTCAQLFTSR | Carboxymethyl (C)[5] |
| 15 | 1480.7366 | 1480.7848 | 0.0482 | 3078 | 3090 | WALMIDPHGQANK |  |
| 16 | 1609.7461 | 1609.6573 | -0.0888 | 2876 | 2889 | NVSSACEGLCKWVR | Carboxymethyl (C)[6] |
| 17 | 1611.8199 | 1611.7551 | -0.0648 | 2560 | 2574 | ISFSLAMSPIGDAFR |  |
| 18 | 1611.8199 | 1611.7551 | -0.0648 | 2560 | 2574 | ISFSLAMSPIGDAFR |  |
| 19 | 1627.8148 | 1627.7557 | -0.0591 | 2560 | 2574 | ISFSLAMSPIGDAFR | Oxidation (M)[7] |
| 20 | 1798.8285 | 1798.9276 | 0.0991 | 1246 | 1259 | WLQQVEQMMLASMR | Oxidation (M)[8,9,13] |
| 21 | 1798.8285 | 1798.9276 | 0.0991 | 1246 | 1259 | WLQQVEQMMLASMR | Oxidation (M)[8,9,13] |
| 22 | 1816.8761 | 1816.9557 | 0.0796 | 3705 | 3718 | ISMWQIQMFLNDYK |  |
| 23 | 1846.9215 | 1846.9594 | 0.0379 | 2952 | 2966 | DLEENIEICSQKLVR | Carboxymethyl (C)[9] |
| 24 | 1848.866 | 1848.9395 | 0.0735 | 3705 | 3718 | ISMWQIQMFLNDYK | Oxidation (M)[3,8] |
| 25 | 1848.866 | 1848.9395 | 0.0735 | 3705 | 3718 | ISMWQIQMFLNDYK | Oxidation (M)[3,8] |
| Peptide Number | Calculated Mass | Observed Mass | Match Error Da | Start Sequence Position | End Sequence Position | Sequence | Modification |
| 26 | 1863.9608 | 1864.0045 | 0.0437 | 1904 | 1919 | FVVQTSPIHLAFSMMR |  |
| 27 | 1864.9515 | 1864.9558 | 0.0043 | 2797 | 2812 | LVMESICIMKGMKPER |  |
| 28 | 1881.964 | 1882.0144 | 0.0504 | 2560 | 2576 | ISFSLAMSPIGDAFRNR |  |
| 29 | 1924.9572 | 1925.0831 | 0.1259 | 1209 | 1225 | LEFTDNLEIVGMISSEK |  |
| 30 | 1945.978 | 1946.089 | 0.111 | 89 | 104 | TSWTLAAPFKEQHHHR |  |
| 31 | 1945.978 | 1946.089 | 0.111 | 89 | 104 | TSWTLAAPFKEQHHHR |  |
| 32 | 1950.9775 | 1950.9614 | -0.0161 | 939 | 955 | QYIPILSISCNPGMKDR | Oxidation (M)[14] |
| 33 | 1983.9739 | 1984.0151 | 0.0412 | 1981 | 1997 | NLIMGMDDNHPRPKSVK | Oxidation (M)[4,6] |
| 34 | 1993.8816 | 1994.0769 | 0.1953 | 1449 | 1466 | QCVVFNCSDGLDYKAMGK | Oxidation (M)[16] |
| 35 | 2123.0596 | 2123.1526 | 0.093 | 2001 | 2017 | NNIFPERGSIYDFYFIK |  |
| 36 | 2295.1116 | 2295.0981 | -0.0135 | 1241 | 1259 | GMVEKWLQQVEQMMLASMR |  |

**Supplementary table 2** Proteomic identification of IgE from a *PGM3* patient


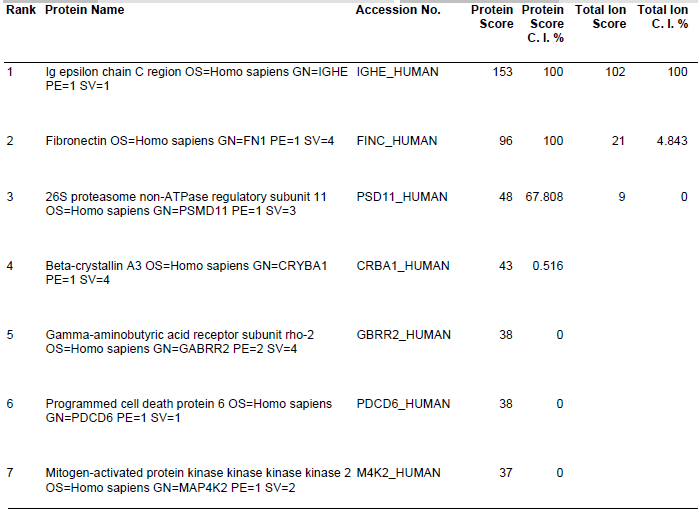


| **Supplementary Table 2a** : IGHE_HUMAN : Ig epsilon chain C region OS=Homo sapiens GN=IGHE PE=1 SV=1 | | | | | | | |
| --- | --- | --- | --- | --- | --- | --- | --- |
|  |  |  |  |  |  |  |  |
| Peptide Number | Calculated Mass | Observed Mass | Match Error Da | Start Sequence Position | End Sequence Position | Sequence | Modification |
| 1 | 813.4002 | 813.4138 | 0.0136 | 184 | 189 | HWLSDR |  |
| 2 | 956.5424 | 956.5602 | 0.0178 | 301 | 308 | VTHPHLPR |  |
| 3 | 1003.4995 | 1003.5162 | 0.0167 | 381 | 389 | GSGFFVFSR |  |
| 4 | 1003.4995 | 1003.5162 | 0.0167 | 381 | 389 | GSGFFVFSR |  |
| 5 | 1457.6002 | 1457.629 | 0.0288 | 290 | 300 | DWIEGETYQCR | Carboxymethyl (C)[10] |
| 6 | 1457.6002 | 1457.629 | 0.0288 | 290 | 300 | DWIEGETYQCR | Carboxymethyl (C)[10] |
| 7 | 1480.7502 | 1480.777 | 0.0268 | 407 | 420 | AVHEAASPSQTVQR |  |
| 8 | 1611.7108 | 1611.74 | 0.0292 | 395 | 406 | AEWEQKDEFICR | Carboxymethyl (C)[11] |
| 9 | 1611.7108 | 1611.74 | 0.0292 | 395 | 406 | AEWEQKDEFICR | Carboxymethyl (C)[11] |
| 10 | 1848.8915 | 1848.9183 | 0.0268 | 322 | 338 | AAPEVYAFATPEWPGSR |  |
| 11 | 1848.8915 | 1848.9183 | 0.0268 | 322 | 338 | AAPEVYAFATPEWPGSR |  |
| 12 | 1925.028 | 1925.0743 | 0.0463 | 216 | 232 | GVSAYLSRPSPFDLFIR |  |
| 13 | 2168.1318 | 2168.0969 | -0.0349 | 249 | 268 | GTVNLTWSRASGKPVNHSTR |  |
| 14 | 2295.0234 | 2295.0784 | 0.055 | 190 | 208 | TYTCQVTYQGHTFEDSTKK | Carboxymethyl (C)[4] |

**Supplementary Table 2b** : FINC_HUMAN : Fibronectin OS=Homo sapiens GN=FN1 PE=1 SV=4

| Peptide Number | Calculated Mass | Observed Mass | Match Error Da | Start Sequence Position | End Sequence Position | Sequence | Modification |
| --- | --- | --- | --- | --- | --- | --- | --- |
| 1 | 1171.4183 | 1171.4355 | 0.0172 | 585 | 592 | YQCYCYGR | Carboxymethyl (C)[3,5] |
| 2 | 1323.7128 | 1323.7386 | 0.0258 | 1117 | 1129 | LGVRPSQGGEAPR |  |
| 3 | 1349.6848 | 1349.7087 | 0.0239 | 1562 | 1573 | WLPSSSPVTGYR |  |
| 4 | 1355.6954 | 1355.71 | 0.0146 | 1881 | 1891 | IYLYTLNDNAR |  |
| 5 | 1356.6669 | 1356.6902 | 0.0233 | 1392 | 1402 | HHPEHFSGRPR |  |
| 6 | 1356.6669 | 1356.6902 | 0.0233 | 1392 | 1402 | HHPEHFSGRPR |  |
| 7 | 1401.6659 | 1401.6877 | 0.0218 | 58 | 67 | HYQINQQWER |  |
| 8 | 1401.6659 | 1401.6877 | 0.0218 | 58 | 67 | HYQINQQWER |  |
| 9 | 1431.7491 | 1431.7711 | 0.022 | 831 | 842 | WSRPQAPITGYR |  |
| 10 | 1431.7491 | 1431.7711 | 0.022 | 831 | 842 | WSRPQAPITGYR |  |
| 11 | 1484.6838 | 1484.7142 | 0.0304 | 504 | 515 | GEWTCIAYSQLR | Carboxymethyl (C)[5] |
| 12 | 1591.8075 | 1591.8226 | 0.0151 | 1525 | 1539 | GDSPASSKPISINYR |  |
| 13 | 1629.8707 | 1629.8994 | 0.0287 | 939 | 953 | VDVIPVNLPGEHGQR |  |
| 14 | 1629.8707 | 1629.8994 | 0.0287 | 939 | 953 | VDVIPVNLPGEHGQR |  |
| 15 | 1727.8058 | 1727.8364 | 0.0306 | 398 | 411 | YSFCTDHTVLVQTR | Carboxymethyl (C)[4] |
| 16 | 1732.948 | 1732.986 | 0.038 | 1055 | 1070 | NLQPASEYTVSLVAIK |  |
| 17 | 1798.767 | 1798.9091 | 0.1421 | 117 | 132 | DSMIWDCTCIGAGRGR | Carboxymethyl (C)[7] |
| 18 | 1807.9048 | 1807.9274 | 0.0226 | 923 | 938 | VTIMWTPPESAVTGYR |  |
| 19 | 1823.8997 | 1823.9342 | 0.0345 | 923 | 938 | VTIMWTPPESAVTGYR | Oxidation (M)[4] |
| 20 | 1848.8843 | 1848.9183 | 0.034 | 253 | 269 | GNLLQCICTGNGRGEWK |  |
| 21 | 1848.8843 | 1848.9183 | 0.034 | 253 | 269 | GNLLQCICTGNGRGEWK |  |
| 22 | 1863.8832 | 1863.9285 | 0.0453 | 273 | 290 | HTSVQTTSSGSGPFTDVR |  |
| 23 | 1876.7523 | 1876.9359 | 0.1836 | 535 | 550 | HEEGHMLNCTCFGQGR | Carboxymethyl (C)[9] |
| 24 | 1926.0483 | 1926.0762 | 0.0279 | 1285 | 1301 | VTWAPPPSIDLTNFLVR |  |
| 25 | 1955.0081 | 1955.0394 | 0.0313 | 1435 | 1452 | EESPLLIGQQSTVSDVPR |  |
| 26 | 1993.9905 | 1994.0485 | 0.058 | 959 | 976 | NTFAEVTGLSPGVTYYFK |  |
| 27 | 2168.0505 | 2168.0969 | 0.0464 | 1480 | 1500 | ITYGETGGNSPVQEFTVPGSK |  |
| 28 | 2470.3188 | 2470.3594 | 0.0406 | 1501 | 1524 | STATISGLKPGVDYTITVYAVTGR |  |
| 29 | 2475.2976 | 2475.3491 | 0.0515 | 1252 | 1274 | DDKESVPISDTIIPAVPPPTDLR |  |
| 30 | 2799.4788 | 2799.532 | 0.0532 | 670 | 694 | GLKPGVVYEGQLISIQQYGHQEVTR |  |
| 31 | 2918.4319 | 2918.4622 | 0.0303 | 1356 | 1382 | TGLDSPTGIDFSDITANSFTVHWIAPR |  |
| 32 | 3042.5894 | 3042.6023 | 0.0129 | 1170 | 1197 | VVTPLSPPTNLHLEANPDTGVLTVSWER |  |

| **Supplementary Table 2c** : PSD11_HUMAN : 26S proteasome non-ATPase regulatory subunit 11 OS=Homo sapiens GN=PSMD11 PE=1 SV=3 | | | | | | | |
| --- | --- | --- | --- | --- | --- | --- | --- |
|  |  |  |  |  |  |  |  |
| Peptide Number | Calculated Mass | Observed Mass | Match Error Da | Start Sequence Position | End Sequence Position | Sequence | Modification |
| 1 | 1091.6129 | 1091.6097 | -0.0032 | 364 | 372 | KLSQMILDK | Oxidation (M)[5] |
| 2 | 1091.6129 | 1091.6097 | -0.0032 | 365 | 373 | LSQMILDKK | Oxidation (M)[4] |
| 3 | 1323.7419 | 1323.7386 | -0.0033 | 72 | 82 | YVRPFLNSISK |  |
| 4 | 1400.7996 | 1400.7682 | -0.0314 | 47 | 59 | EQSILELGSLLAK |  |
| 5 | 1457.7743 | 1457.629 | -0.1453 | 247 | 258 | AITSLKYMLLCK | Oxidation (M)[8], Carboxymethyl (C)[11] |
| 6 | 1457.7743 | 1457.629 | -0.1453 | 247 | 258 | AITSLKYMLLCK | Oxidation (M)[8], Carboxymethyl (C)[11] |
| 7 | 1480.8481 | 1480.777 | -0.0711 | 21 | 33 | EASIDILHSIVKR |  |
| 8 | 1848.9636 | 1848.9183 | -0.0453 | 188 | 205 | AALTSARTTANAIYCPPK |  |
| 9 | 1848.9636 | 1848.9183 | -0.0453 | 188 | 205 | AALTSARTTANAIYCPPK |  |
| 10 | 1954.9902 | 1955.0394 | 0.0492 | 206 | 223 | LQATLDMQSGIIHAAEEK |  |
| 11 | 2168.2107 | 2168.0969 | -0.1138 | 259 | 278 | IMLNTPEDVQALVSGKLALR |  |

| **Supplementary Table 2d** : CRBA1_HUMAN : Beta-crystallin A3 OS=Homo sapiens GN=CRYBA1 PE=1 SV=4 | | | | | | | |
| --- | --- | --- | --- | --- | --- | --- | --- |
|  |  |  |  |  |  |  |  |
| Peptide Number | Calculated Mass | Observed Mass | Match Error Da | Start Sequence Position | End Sequence Position | Sequence | Modification |
| 1 | 1455.7114 | 1455.6382 | -0.0732 | 33 | 44 | ITIYDQENFQGK |  |
| 2 | 1484.7566 | 1484.7142 | -0.0424 | 126 | 137 | MTIFEKENFIGR |  |
| 3 | 1611.8125 | 1611.74 | -0.0725 | 33 | 45 | ITIYDQENFQGKR |  |
| 4 | 1611.8125 | 1611.74 | -0.0725 | 33 | 45 | ITIYDQENFQGKR |  |
| 5 | 1727.8459 | 1727.8364 | -0.0095 | 197 | 211 | EWGSHAQTSQIQSIR |  |
| 6 | 1848.8374 | 1848.9183 | 0.0809 | 163 | 177 | IQSGAWVCYQYPGYR | Carboxymethyl (C)[8] |
| 7 | 1848.8374 | 1848.9183 | 0.0809 | 163 | 177 | IQSGAWVCYQYPGYR | Carboxymethyl (C)[8] |
| 8 | 1863.9204 | 1863.9285 | 0.0081 | 110 | 125 | LMSFRPICSANHKESK | Oxidation (M)[2] |
| 9 | 1883.9471 | 1884.0398 | 0.0927 | 197 | 212 | EWGSHAQTSQIQSIRR |  |

| **Supplementary Table 2e** : GBRR2_HUMAN : Gamma-aminobutyric acid receptor subunit rho-2 OS=Homo sapiens GN=GABRR2 PE=2 SV=4 | | | | | | | |
| --- | --- | --- | --- | --- | --- | --- | --- |
|  |  |  |  |  |  |  |  |
| Peptide Number | Calculated Mass | Observed Mass | Match Error Da | Start Sequence Position | End Sequence Position | Sequence | Modification |
| 1 | 813.3559 | 813.4138 | 0.0579 | 147 | 153 | SMTFDGR |  |
| 2 | 1473.7889 | 1473.6633 | -0.1256 | 158 | 169 | IWVPDVFFVHSK |  |
| 3 | 1484.734 | 1484.7142 | -0.0198 | 420 | 431 | SHILTEEERQDK |  |
| 4 | 1592.818 | 1592.855 | 0.037 | 459 | 471 | IFQNTHAIDKYSR |  |
| 5 | 1629.89 | 1629.8994 | 0.0094 | 158 | 170 | IWVPDVFFVHSKR |  |
| 6 | 1629.89 | 1629.8994 | 0.0094 | 158 | 170 | IWVPDVFFVHSKR |  |
| 7 | 1807.8756 | 1807.9274 | 0.0518 | 170 | 184 | RSFTHDTTTDNIMLR |  |
| 8 | 1816.905 | 1816.9424 | 0.0374 | 53 | 67 | WTGQVEMPKPSHLYK | Oxidation (M)[7] |
| 9 | 1823.8705 | 1823.9342 | 0.0637 | 170 | 184 | RSFTHDTTTDNIMLR | Oxidation (M)[13] |
| 10 | 1854.0267 | 1853.9614 | -0.0653 | 32 | 46 | LILFLFCLMVLVESR | Carboxymethyl (C)[7] |
| 11 | 2294.9905 | 2295.0784 | 0.0879 | 399 | 419 | TMMLDGSYSESEANSLAGYPR | Oxidation (M)[2] |
| 12 | 2799.3303 | 2799.532 | 0.2017 | 1 | 25 | MVKPGGICSATGYWKAAFCLTDVHK | Carboxymethyl (C)[8,19] |

| **Supplementary Table 2f** : PDCD6_HUMAN : Programmed cell death protein 6 OS=Homo sapiens GN=PDCD6 PE=1 SV=1 | | | | | | |  |
| --- | --- | --- | --- | --- | --- | --- | --- |
|  |  |  |  |  |  |  |  |
| Peptide Number | Calculated Mass | Observed Mass | Match Error Da | Start Sequence Position | End Sequence Position | Sequence | Modification |
| 1 | 1355.6624 | 1355.71 | 0.0476 | 67 | 77 | SIISMFDRENK | Oxidation (M)[5] |
| 2 | 1356.7271 | 1356.6902 | -0.0369 | 126 | 136 | LSDQFHDILIR |  |
| 3 | 1356.7271 | 1356.6902 | -0.0369 | 126 | 136 | LSDQFHDILIR |  |
| 4 | 1414.6267 | 1414.6902 | 0.0635 | 101 | 112 | TYDRDNSGMIDK |  |
| 5 | 1484.822 | 1484.7142 | -0.1078 | 126 | 137 | LSDQFHDILIRK |  |
| 6 | 1876.8977 | 1876.9359 | 0.0382 | 91 | 104 | YITDWQNVFRTYDR |  |
| 7 | 3042.4189 | 3042.6023 | 0.1834 | 167 | 191 | RYDTDQDGWIQVSYEQYLSMVFSIV |  |

| **Supplementary Table 2g** : M4K2_HUMAN : Mitogen-activated protein kinase kinase kinase kinase 2 OS=Homo sapiens GN=MAP4K2 PE=1 SV=2 | | | | | | | |
| --- | --- | --- | --- | --- | --- | --- | --- |
|  |  |  |  |  |  |  |  |
| Peptide Number | Calculated Mass | Observed Mass | Match Error Da | Start Sequence Position | End Sequence Position | Sequence | Modification |
| 1 | 1053.4492 | 1053.4166 | -0.0326 | 468 | 476 | VHMGACFSK | Oxidation (M)[3], Carboxymethyl (C)[6] |
| 2 | 1091.5448 | 1091.6097 | 0.0649 | 597 | 605 | GCLQCRVVR | Carboxymethyl (C)[2] |
| 3 | 1091.5448 | 1091.6097 | 0.0649 | 597 | 605 | GCLQCRVVR | Carboxymethyl (C)[2] |
| 4 | 1349.6188 | 1349.7087 | 0.0899 | 592 | 602 | IPDTKGCLQCR | Carboxymethyl (C)[7,10] |
| 5 | 1356.6907 | 1356.6902 | -0.0005 | 22 | 34 | VGAGTYGDVYKAR |  |
| 6 | 1356.6907 | 1356.6902 | -0.0005 | 22 | 34 | VGAGTYGDVYKAR |  |
| 7 | 1807.828 | 1807.9274 | 0.0994 | 452 | 467 | EDPERSSCHGLPPTPK | Carboxymethyl (C)[8] |
| 8 | 1876.9486 | 1876.9359 | -0.0127 | 67 | 82 | ECRHPNVVAYIGSYLR |  |
| 9 | 1881.8921 | 1881.9828 | 0.0907 | 468 | 484 | VHMGACFSKVFNGCPLR | Oxidation (M)[3] |
| 10 | 1884.028 | 1884.0398 | 0.0118 | 242 | 256 | WTQNFHHFLKLALTK |  |
| 11 | 2168.0845 | 2168.0969 | 0.0124 | 170 | 188 | SFIGTPYWMAPEVAAVERK | Oxidation (M)[9] |
| 12 | 2799.5364 | 2799.532 | -0.0044 | 634 | 659 | FLLLKNFSSPLPSPAGMLEPLVLDGK | Oxidation (M)[17] |

**Supplementary table 3** Summary of observed glycosylation at Asn21 of IgE from a *PGM3* patient (Figure 2)

| Observed m/z | Charge State | Theoretical Observed m/z | Delta Mass (Da) | Observed Mass (M) | Peptide Sequence | Protease Used | Modifications | Elution Time (min) | \| Observed Glycan \| \| --- \| |
| --- | --- | --- | --- | --- | --- | --- | --- | --- | --- | --- |
| 1198.2 | 3 | 1198.2 | 0.03 | 3591.6 | TRCCKNIPSNATSVTL | Chymotrypsin | Carboxymethyl-Cys (3,4) | 30.5-31.5 | 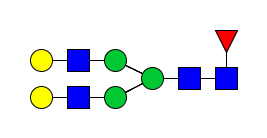 |
| 1265.9 | 3 | 1265.9 | 0.03 | 3794.7 | TRCCKNIPSNATSVTL | Chymotrypsin | Carboxymethyl-Cys (3,4) | 30.5-31.5 | 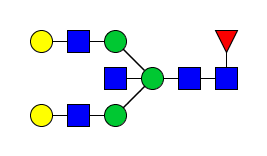 |
| 971.6 | 4 | 971.7 | -0.06 | 3882.4 | TRCCKNIPSNATSVTL | Chymotrypsin | Carboxymethyl-Cys (3,4) | 32.5-33.5 | 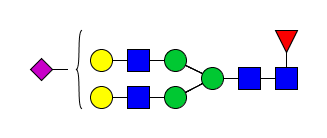 |
| 1022.4 | 4 | 1022.4 | -0.03 | 4085.6 | TRCCKNIPSNATSVTL | Chymotrypsin | Carboxymethyl-Cys (3,4) | 32.5-33.5 | 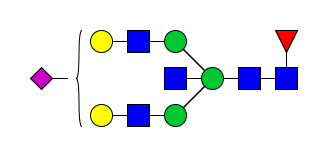 |
| 1198.1 | 3 | 1198.2 | -0.07 | 3591.3 | TRCCKNIPSNATSVTL | Chymotrypsin | Carboxymethyl-Cys (3,4) | 32.5-33.5 | 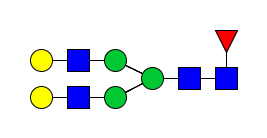 |
| 1265.8 | 3 | 1265.9 | -0.07 | 3794.4 | TRCCKNIPSNATSVTL | Chymotrypsin | Carboxymethyl-Cys (3,4) | 32.5-33.5 | 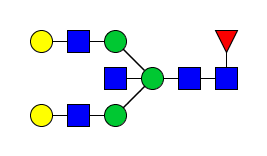 |
| 1295.1 | 3 | 1295.2 | -0.10 | 3882.3 | TRCCKNIPSNATSVTL | Chymotrypsin | Carboxymethyl-Cys (3,4) | 32.5-33.5 | 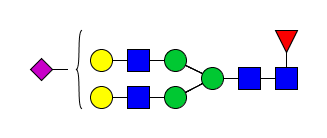 |
| 1362.9 | 3 | 1362.9 | 0.00 | 4085.7 | TRCCKNIPSNATSVTL | Chymotrypsin | Carboxymethyl-Cys (3,4) | 32.5-33.5 | 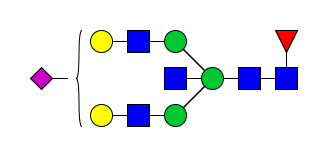 |
| 1533.1 | 2 | 1533.2 | -0.06 | 3064.2 | TRCCKNIPSNATSVTL | Chymotrypsin | Carboxymethyl-Cys (3,4) | 32.5-33.5 | 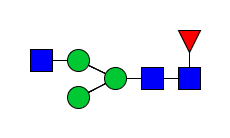 |
| 1614.1 | 2 | 1614.2 | -0.09 | 3226.2 | TRCCKNIPSNATSVTL | Chymotrypsin | Carboxymethyl-Cys (3,4) | 32.5-33.5 | 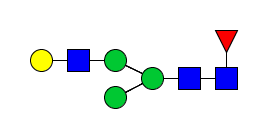 |
| 1715.6 | 2 | 1715.7 | -0.13 | 3429.2 | TRCCKNIPSNATSVTL | Chymotrypsin | Carboxymethyl-Cys (3,4) | 32.5-33.5 | 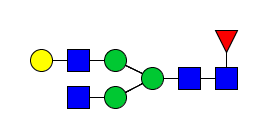 |
| 1796.7 | 2 | 1796.8 | -0.06 | 3591.4 | TRCCKNIPSNATSVTL | Chymotrypsin | Carboxymethyl-Cys (3,4) | 32.5-33.5 | 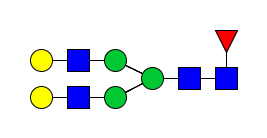 |
| 1942.2 | 2 | 1942.3 | -0.10 | 3882.4 | TRCCKNIPSNATSVTL | Chymotrypsin | Carboxymethyl-Cys (3,4) | 32.5-33.5 | 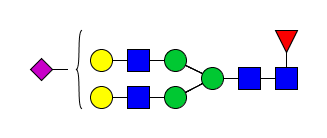 |
| 2043.7 | 2 | 2043.8 | -0.14 | 4085.4 | TRCCKNIPSNATSVTL | Chymotrypsin | Carboxymethyl-Cys (3,4) | 32.5-33.5 | 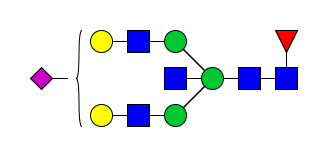 |
| 1044.4 | 4 | 1044.4 | -0.03 | 4173.6 | TRCCKNIPSNATSVTL | Chymotrypsin | Carboxymethyl-Cys (3,4) | 35.0-37.0 | 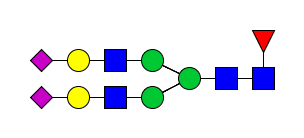 |
| 1095.1 | 4 | 1095.2 | -0.10 | 4376.4 | TRCCKNIPSNATSVTL | Chymotrypsin | Carboxymethyl-Cys (3,4) | 35.0-37.0 | 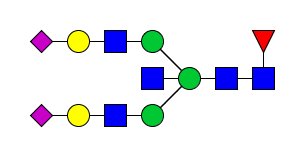 |
| 1241.1 | 3 | 1241.2 | -0.09 | 3720.3 | TRCCKNIPSNATSVTL | Chymotrypsin | Carboxymethyl-Cys (3,4) | 35.0-37.0 | 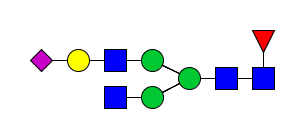 |
| 1362.9 | 3 | 1362.9 | 0.00 | 4085.7 | TRCCKNIPSNATSVTL | Chymotrypsin | Carboxymethyl-Cys (3,4) | 35.0-37.0 | 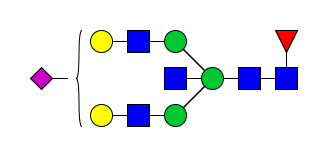 |
| 1392.2 | 3 | 1392.2 | -0.04 | 4173.6 | TRCCKNIPSNATSVTL | Chymotrypsin | Carboxymethyl-Cys (3,4) | 35.0-37.0 | 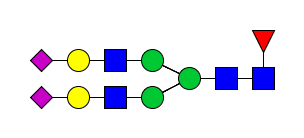 |
| 1459.9 | 3 | 1459.9 | -0.03 | 4376.7 | TRCCKNIPSNATSVTL | Chymotrypsin | Carboxymethyl-Cys (3,4) | 35.0-37.0 | 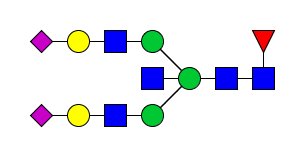 |

**Supplementary table 4** Summary of observed glycosylation at Asn49 of IgE from a *PGM3* patient (Figure 3)

| Observed m/z | Charge State | Theoretical Observed m/z | Delta Mass (Da) | Observed Mass (M) | Peptide Sequence | Protease Used | Modifications | Elution Time (min) | \| Glycan Observed \| \| --- \| |
| --- | --- | --- | --- | --- | --- | --- | --- | --- | --- | --- |
| 1160.2 | 3 | 1160.2 | 0.04 | 3477.6 | DTGSLNGTTMTLPATTL | Chymotrypsin | Oxidation (M), 10 | 37.0-38.0 | 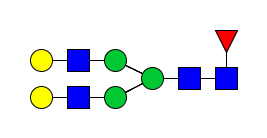 |
| 1227.8 | 3 | 1227.9 | -0.05 | 3680.4 | DTGSLNGTTMTLPATTL | Chymotrypsin | Oxidation (M), 10 | 37.0-38.0 | 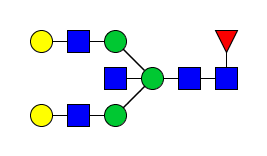 |
| 1257.2 | 3 | 1257.2 | 0.01 | 3768.6 | DTGSLNGTTMTLPATTL | Chymotrypsin | Oxidation (M), 10 | 40.0-42.0 | 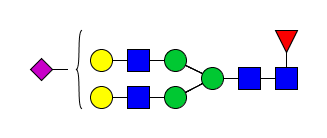 |
| 1324.9 | 3 | 1324.9 | 0.01 | 3971.7 | DTGSLNGTTMTLPATTL | Chymotrypsin | Oxidation (M), 10 | 40.0-42.0 | 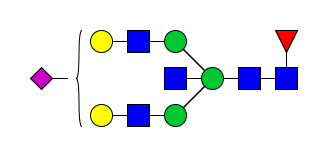 |
| 1658.6 | 2 | 1658.7 | -0.11 | 3315.2 | DTGSLNGTTMTLPATTL | Chymotrypsin | Oxidation (M), 10 | 40.0-42.0 | 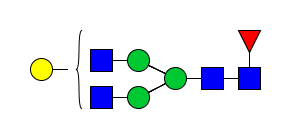 |
| 1804.1 | 2 | 1804.3 | -0.16 | 3606.2 | DTGSLNGTTMTLPATTL | Chymotrypsin | Oxidation (M), 10 | 40.0-42.0 | 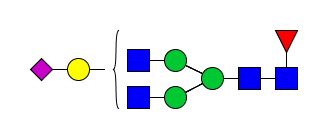 |
| 1885.2 | 2 | 1885.3 | -0.08 | 3768.4 | DTGSLNGTTMTLPATTL | Chymotrypsin | Oxidation (M), 10 | 40.0-42.0 | 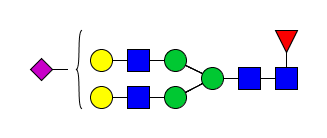 |
| 1986.6 | 2 | 1986.8 | -0.22 | 3971.2 | DTGSLNGTTMTLPATTL | Chymotrypsin | Oxidation (M), 10 | 40.0-42.0 | 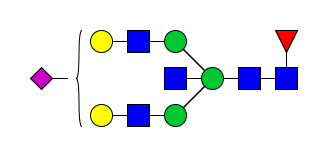 |
| 1015.9 | 4 | 1015.9 | -0.02 | 4059.6 | DTGSLNGTTMTLPATTL | Chymotrypsin | Oxidation (M), 10 | 46.0-47.5 | 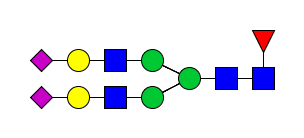 |
| 1066.6 | 4 | 1066.7 | -0.09 | 4262.4 | DTGSLNGTTMTLPATTL | Chymotrypsin | Oxidation (M), 10 | 46.0-47.5 | 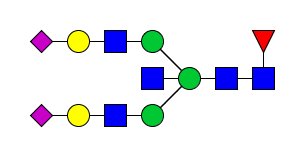 |
| 1257.1 | 3 | 1257.2 | -0.09 | 3768.3 | DTGSLNGTTMTLPATTL | Chymotrypsin | Oxidation (M), 10 | 46.0-47.5 | 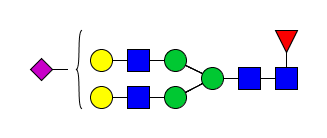 |
| 1354.2 | 3 | 1354.2 | -0.02 | 4059.6 | DTGSLNGTTMTLPATTL | Chymotrypsin | Oxidation (M), 10 | 46.0-47.5 | 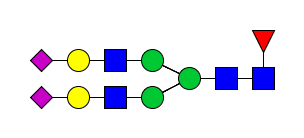 |
| 1421.8 | 3 | 1421.9 | -0.12 | 4262.4 | DTGSLNGTTMTLPATTL | Chymotrypsin | Oxidation (M), 10 | 46.0-47.5 | 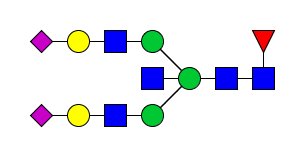 |
| 1702.6 | 2 | 1702.7 | -0.12 | 3403.2 | DTGSLNGTTMTLPATTL | Chymotrypsin | Oxidation (M), 10 | 46.0-47.5 | 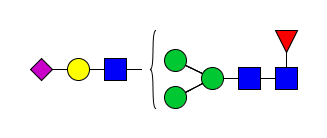 |
| 1804.2 | 2 | 1804.3 | -0.06 | 3606.4 | DTGSLNGTTMTLPATTL | Chymotrypsin | Oxidation (M), 10 | 46.0-47.5 | 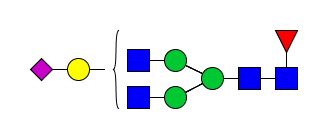 |
| 2030.7 | 2 | 2030.8 | -0.13 | 4059.4 | DTGSLNGTTMTLPATTL | Chymotrypsin | Oxidation (M), 10 | 46.0-47.5 | 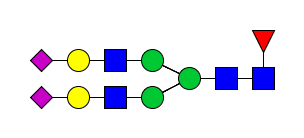 |

**Supplementary table 5** Summary of observed glycosylation at Asn99 of IgE from a *PGM3* patient (Figure 4)

| Observed m/z | Charge State | Theoretical Observed m/z | Delta Mass (Da) | Observed Mass (M) | Peptide Sequence | Protease Used | Modifications | Elution Time (min) | Glycan Observed |
| --- | --- | --- | --- | --- | --- | --- | --- | --- | --- |
| 987.4 | 3 | 987.4 | -0.02 | 2959.2 | VAHTPSSTDWVDNK | Trypsin | n/a | 21.0-23.0 | 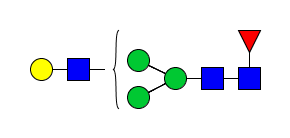 |
| 996.2 | 4 | 996.2 | 0.04 | 3980.8 | VAHTPSSTDWVDNK | Trypsin | n/a | 21.0-23.0 | 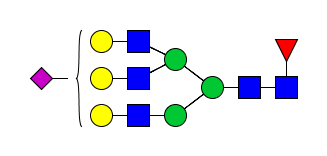 |
| 1028.4 | 4 | 1028.4 | -0.02 | 4109.6 | VAHTPSSTDWVDNK | Trypsin | n/a | 21.0-23.0 | 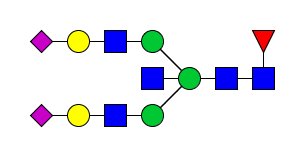 |
| 1055.1 | 3 | 1055.1 | -0.01 | 3162.3 | VAHTPSSTDWVDNK | Trypsin | n/a | 21.0-23.0 | 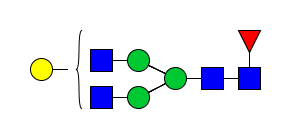 |
| 1068.9 | 3 | 1068.8 | 0.11 | 3203.7 | VAHTPSSTDWVDNK | Trypsin | n/a | 21.0-23.0 | 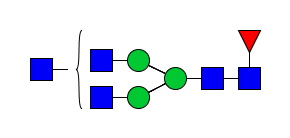 |
| 1109.1 | 3 | 1109.1 | -0.03 | 3324.3 | VAHTPSSTDWVDNK | Trypsin | n/a | 21.0-23.0 | 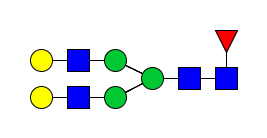 |
| 1157.4 | 3 | 1157.5 | -0.08 | 3469.2 | VAHTPSSTDWVDNK | Trypsin | n/a | 21.0-23.0 | 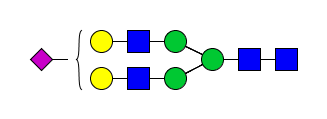 |
| 1176.8 | 3 | 1176.8 | -0.02 | 3527.4 | VAHTPSSTDWVDNK | Trypsin | n/a | 21.0-23.0 | 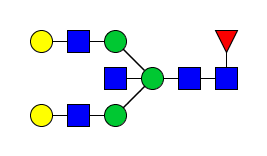 |
| 1206.1 | 3 | 1206.2 | -0.06 | 3615.3 | VAHTPSSTDWVDNK | Trypsin | n/a | 21.0-23.0 | 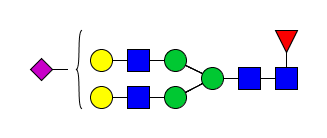 |
| 1273.8 | 3 | 1273.9 | -0.05 | 3818.4 | VAHTPSSTDWVDNK | Trypsin | n/a | 21.0-23.0 | 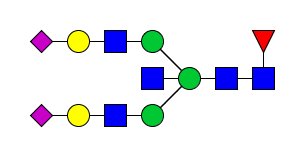 |
| 1298.1 | 2 | 1298.1 | 0.04 | 2594.2 | VAHTPSSTDWVDNK | Trypsin | n/a | 21.0-23.0 | 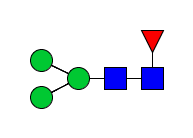 |
| 1303.2 | 3 | 1303.2 | 0.01 | 3906.6 | VAHTPSSTDWVDNK | Trypsin | n/a | 21.0-23.0 | 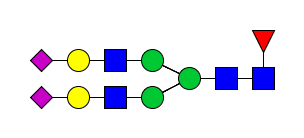 |
| 1327.9 | 3 | 1327.9 | 0.03 | 3980.7 | VAHTPSSTDWVDNK | Trypsin | n/a | 21.0-23.0 | 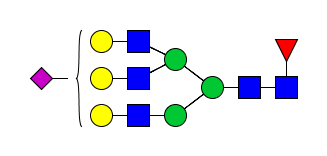 |
| 1370.8 | 3 | 1370.9 | -0.09 | 4109.4 | VAHTPSSTDWVDNK | Trypsin | n/a | 21.0-23.0 | 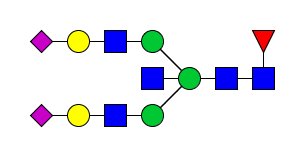 |
| 1399.6 | 2 | 1399.6 | 0.00 | 2797.2 | VAHTPSSTDWVDNK | Trypsin | n/a | 21.0-23.0 | 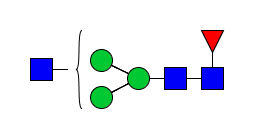 |
| 1407.6 | 2 | 1407.6 | 0.00 | 2813.2 | VAHTPSSTDWVDNK | Trypsin | n/a | 21.0-23.0 | 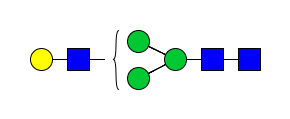 |
| 1424.9 | 3 | 1424.9 | 0.00 | 4271.7 | VAHTPSSTDWVDNK | Trypsin | n/a | 21.0-23.0 | 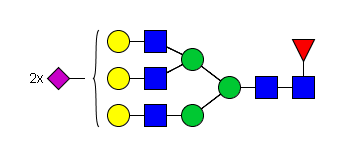 |
| 1480.6 | 2 | 1480.6 | -0.02 | 2959.2 | VAHTPSSTDWVDNK | Trypsin | n/a | 21.0-23.0 | 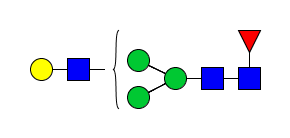 |
| 1545.2 | 2 | 1545.1 | 0.05 | 3088.4 | VAHTPSSTDWVDNK | Trypsin | n/a | 21.0-23.0 | 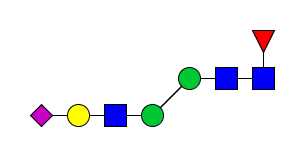 |
| 1582.2 | 2 | 1582.2 | 0.04 | 3162.4 | VAHTPSSTDWVDNK | Trypsin | n/a | 21.0-23.0 | 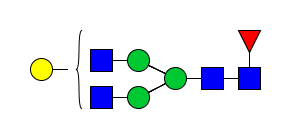 |
| 1626.2 | 2 | 1626.2 | 0.03 | 3250.4 | VAHTPSSTDWVDNK | Trypsin | n/a | 21.0-23.0 | 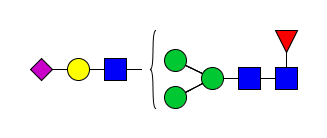 |
| 1663.2 | 2 | 1663.2 | 0.01 | 3324.4 | VAHTPSSTDWVDNK | Trypsin | n/a | 21.0-23.0 | 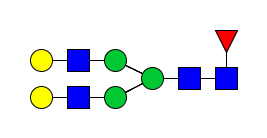 |
| 1727.7 | 2 | 1727.7 | -0.01 | 3453.4 | VAHTPSSTDWVDNK | Trypsin | n/a | 21.0-23.0 | 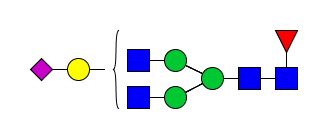 |
| 1808.7 | 2 | 1808.7 | -0.04 | 3615.4 | VAHTPSSTDWVDNK | Trypsin | n/a | 21.0-23.0 | 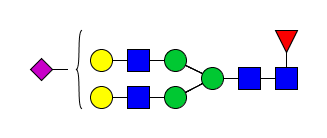 |
| 1954.2 | 2 | 1954.3 | -0.09 | 3906.4 | VAHTPSSTDWVDNK | Trypsin | n/a | 21.0-23.0 | 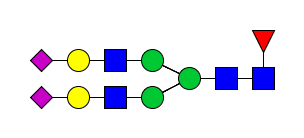 |

**Supplementary table 6** Summary of observed glycosylation at Asn252 and Asn275 of IgE from a *PGM3* patient (Figure 5)

| **Observed m/z** | **Charge State** | **Theoretical Observed m/z** | **Delta Mass (Da)** | **Observed Mass (M)** | **Peptide Sequence** | **Protease Used** | **Modifications** | **Elution Time (min)** | **Glycan Observed** |
| --- | --- | --- | --- | --- | --- | --- | --- | --- | --- |
| 749.7 | 3 | 749.7 | 0.00 | 2246.1 | NGTLTVTSTLPVGTR | Trypsin | n/a | 27.5-32.0 | 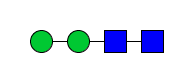 |
| 803.7 | 3 | 803.7 | -0.02 | 2408.1 | NGTLTVTSTLPVGTR | Trypsin | n/a | 27.5-32.0 | 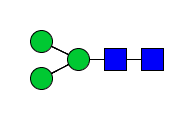 |
| 857.7 | 3 | 857.7 | -0.04 | 2570.1 | NGTLTVTSTLPVGTR | Trypsin | n/a | 27.5-32.0 | 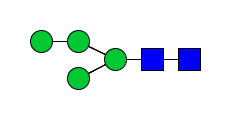 |
| 860.4 | 2 | 860.5 | -0.06 | 1718.8 | NGTLTVTSTLPVGTR | Trypsin | n/a | 27.5-32.0 | 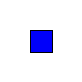 |
| 911.8 | 3 | 911.8 | 0.04 | 2732.4 | NGTLTVTSTLPVGTR | Trypsin | n/a | 27.5-32.0 | 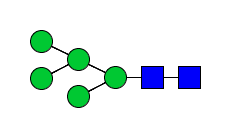 |
| 962 | 2 | 962.0 | 0.00 | 1922 | NGTLTVTSTLPVGTR | Trypsin | n/a | 27.5-32.0 | 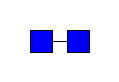 |
| 965.8 | 3 | 965.8 | 0.02 | 2894.4 | NGTLTVTSTLPVGTR | Trypsin | n/a | 27.5-32.0 | 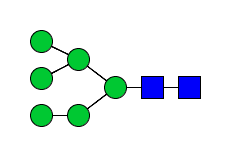 |
| 983 | 3 | 983.4 | -0.42 | 2946 | GTVNLTWSR | Trypsin | n/a | 27.5-32.0 | 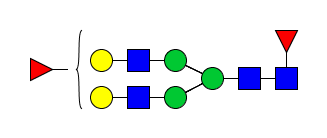 |
| 1002.4 | 3 | 1002.4 | -0.03 | 3004.2 | GTVNLTWSR | Trypsin | n/a | 27.5-32.0 | 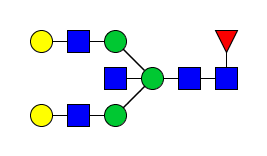 |
| 1019.8 | 3 | 1019.8 | 0.01 | 3056.4 | NGTLTVTSTLPVGTR | Trypsin | n/a | 27.5-32.0 | 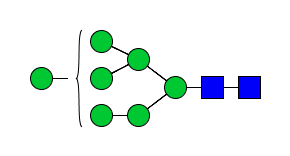 |
| 1031.7 | 3 | 1031.8 | -0.06 | 3092.1 | GTVNLTWSR | Trypsin | n/a | 27.5-32.0 | 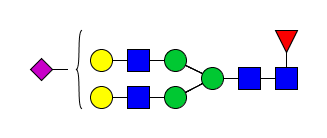 |
| 1043 | 2 | 1043.0 | -0.03 | 2084 | NGTLTVTSTLPVGTR | Trypsin | n/a | 27.5-32.0 | 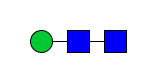 |
| 1073.8 | 3 | 1073.8 | -0.01 | 3218.4 | NGTLTVTSTLPVGTR | Trypsin | n/a | 27.5-32.0 | 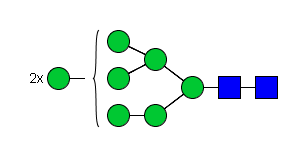 |
| 1099.4 | 3 | 1099.5 | -0.06 | 3295.2 | GTVNLTWSR | Trypsin | n/a | 27.5-32.0 | 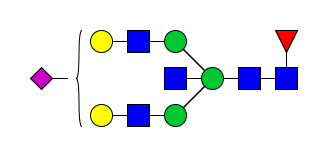 |
| 1124.1 | 2 | 1124.1 | 0.05 | 2246.2 | NGTLTVTSTLPVGTR | Trypsin | n/a | 27.5-32.0 | 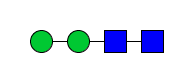 |
| 1127.8 | 3 | 1127.8 | -0.03 | 3380.4 | NGTLTVTSTLPVGTR | Trypsin | n/a | 27.5-32.0 | 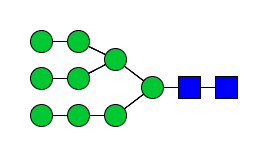 |
| 1153.5 | 3 | 1153.5 | 0.02 | 3457.5 | GTVNLTWSR | Trypsin | n/a | 27.5-32.0 | 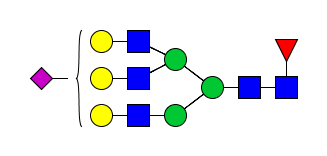 |
| 1205.1 | 2 | 1205.1 | 0.02 | 2408.2 | NGTLTVTSTLPVGTR | Trypsin | n/a | 27.5-32.0 | 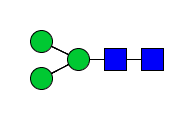 |
| 1219 | 2 | 1219.0 | -0.03 | 2436 | GTVNLTWSR | Trypsin | n/a | 27.5-32.0 | 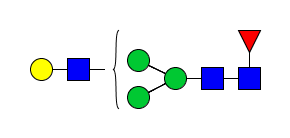 |
| 1239.5 | 2 | 1239.5 | -0.04 | 2477 | GTVNLTWSR | Trypsin | n/a | 27.5-32.0 | 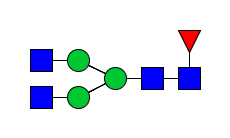 |
| 1286.1 | 2 | 1286.1 | -0.01 | 2570.2 | NGTLTVTSTLPVGTR | Trypsin | n/a | 27.5-32.0 | 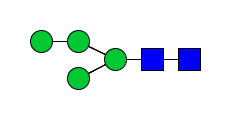 |
| 1320.6 | 2 | 1320.6 | 0.03 | 2639.2 | GTVNLTWSR | Trypsin | n/a | 27.5-32.0 | 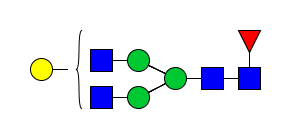 |
| 1367.1 | 2 | 1367.1 | -0.03 | 2732.2 | NGTLTVTSTLPVGTR | Trypsin | n/a | 27.5-32.0 | 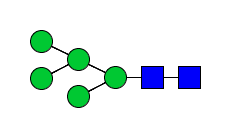 |
| 1401.6 | 2 | 1401.6 | 0.00 | 2801.2 | GTVNLTWSR | Trypsin | n/a | 27.5-32.0 | 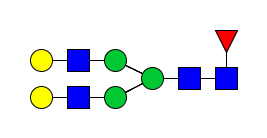 |
| 1448.1 | 2 | 1448.2 | -0.06 | 2894.2 | NGTLTVTSTLPVGTR | Trypsin | n/a | 27.5-32.0 | 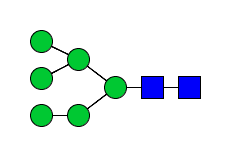 |
| 1466.1 | 2 | 1466.1 | -0.02 | 2930.2 | GTVNLTWSR | Trypsin | n/a | 27.5-32.0 | 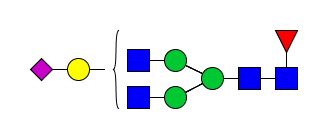 |
| 1503.1 | 2 | 1503.1 | -0.03 | 3004.2 | GTVNLTWSR | Trypsin | n/a | 27.5-32.0 | 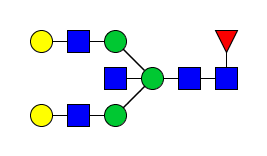 |
| 1516.8 | 1 | 1516.8 | -0.03 | 1515.8 | NGTLTVTSTLPVGTR | Trypsin | n/a | 27.5-32.0 | None |
| 1529.1 | 2 | 1529.2 | -0.09 | 3056.2 | NGTLTVTSTLPVGTR | Trypsin | n/a | 27.5-32.0 | 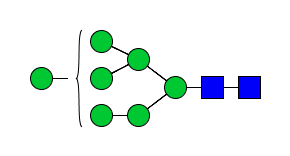 |
| 1547.1 | 2 | 1547.1 | -0.04 | 3092.2 | GTVNLTWSR | Trypsin | n/a | 27.5-32.0 |  |
| 1610.1 | 2 | 1610.2 | -0.11 | 3218.2 | NGTLTVTSTLPVGTR | Trypsin | n/a | 27.5-32.0 |  |
| 1648.7 | 2 | 1648.7 | 0.02 | 3295.4 | GTVNLTWSR | Trypsin | n/a | 27.5-32.0 |  |
| 1691.2 | 2 | 1691.2 | -0.04 | 3380.4 | NGTLTVTSTLPVGTR | Trypsin | n/a | 27.5-32.0 |  |
| 1719.8 | 1 | 1719.9 | -0.11 | 1718.8 | NGTLTVTSTLPVGTR | Trypsin | n/a | 27.5-32.0 |  |

**Supplementary table 7** Summary of observed glycosylation at Asn252 of IgE from a *PGM3* patient (Figure 6)

| **Observed m/z** | **Charge State** | **Theoretical Observed m/z** | **Delta Mass (Da)** | **Observed Mass (M)** | **Peptide Sequence** | **Protease Used** | **Modifications** | **Elution Time (min)** | **Glycan Observed** |
| --- | --- | --- | --- | --- | --- | --- | --- | --- | --- |
| 1099.4 | 3 | 1099.5 | -0.06 | 3295.2 | GTVNLTWSR | Trypsin | n/a | 34.5-36.5 |  |
| 1128.8 | 3 | 1128.8 | 0.00 | 3383.4 | GTVNLTWSR | Trypsin | n/a | 34.5-36.5 |  |
| 1196.4 | 3 | 1196.5 | -0.09 | 3586.2 | GTVNLTWSR | Trypsin | n/a | 34.5-36.5 |  |
| 1239.5 | 2 | 1239.5 | -0.04 | 2477 | GTVNLTWSR | Trypsin | n/a | 34.5-36.5 |  |
| 1283.6 | 2 | 1283.6 | 0.05 | 2565.2 | GTVNLTWSR | Trypsin | n/a | 34.5-36.5 |  |
| 1320.6 | 2 | 1320.6 | 0.03 | 2639.2 | GTVNLTWSR | Trypsin | n/a | 34.5-36.5 |  |
| 1364.6 | 2 | 1364.6 | 0.02 | 2727.2 | GTVNLTWSR | Trypsin | n/a | 34.5-36.5 |  |
| 1466.1 | 2 | 1466.1 | -0.02 | 2930.2 | GTVNLTWSR | Trypsin | n/a | 34.5-36.5 |  |
| 1547.1 | 2 | 1547.1 | -0.04 | 3092.2 | GTVNLTWSR | Trypsin | n/a | 34.5-36.5 |  |
| 1648.7 | 2 | 1648.7 | 0.02 | 3295.4 | GTVNLTWSR | Trypsin | n/a | 34.5-36.5 |  |
| 1692.7 | 2 | 1692.7 | 0.01 | 3383.4 | GTVNLTWSR | Trypsin | n/a | 34.5-36.5 |  |
| 1794.2 | 2 | 1794.2 | -0.03 | 3586.4 | GTVNLTWSR | Trypsin | n/a | 34.5-36.5 |  |

**Supplementary Table 8** Non-glycosylated IgE peptides identified in a trypsin digest of IgE from a *PGM3* patient

| Match to: **gi\|119512** Score: **771** | | | |  | |  | |  | |  | |  |
| --- | --- | --- | --- | --- | --- | --- | --- | --- | --- | --- | --- | --- |
| **RecName: Full=Ig epsilon chain C region** | | | |  | |  | |  | |  | |  |
| Sequence Coverage: **39%** | |  | |  | |  | |  | |  | |  |
| Fixed modifications: Carboxymethyl (C) | | | |  | |  | |  | |  | |  |
| Variable modifications: Oxidation (M) | | | |  | |  | |  | |  | |  |
| Cleavage by Trypsin: cuts C-term side of KR unless next residue is P | | | | | | | |  | |  | |  |
|  |  |  | |  | |  | |  | |  | |  |
| Matched peptides shown in **Bold Red** | | | | | |  | |  | |  | |  |
|  |  |  | |  | |  | |  | |  | |  |
| **1** ASTQSPSVFP LTRCCKNIPS NATSVTLGCL ATGYFPEPVM VTWDTGSLNG | | | | | | | | | | | |  |
| **51** TTMTLPATTL TLSGHYATIS LLTVSGAWAK **QMFTCRVAHT PSSTDWVDNK** | | | | | | | | | |  | |  |
| **101 TFSVCSR**DFT PPTVKILQSS CDGGGHFPPT IQLLCLVSGY TPGTINITWL | | | | | | | | | |  | |  |
| **151** EDGQVMDVDL STASTTQEGE LASTQSELTL SQKHWLSDR**T YTCQVTYQGH** | | | | | | | |  | |  | |  |
| **201 TFEDSTKKCA DSNPR**GVSAY LSRPSPFDLF IR**KSPTITCL VVDLAPSKGT** | | | | | | | |  | |  | |  |
| **251 VNLTWSR**ASG KPVNHSTRKE EKQR**NGTLTV TSTLPVGTRD WIEGETYQCR** | | | | | | | |  | |  | |  |
| **301** VTHPHLPRAL MRSTTKTSGP R**AAPEVYAFA TPEWPGSR**DK RTLACLIQNF | | | | | | | |  | |  | |  |
| **351** MPEDISVQWL HNEVQLPDAR **HSTTQPR**KTK **GSGFFVFSR**L EVTR**AEWEQK** | | | | | | | | |  | |  | |
| **401 DEFICRAVHE AASPSQTVQR AVSVNPGK** |  | |  | |  | |  | |  | |  | |

| Peptide Number | Observed | Calculated Theoretical Mass | Observed Mass (calculated) | Match Error Da | Start Sequence Position | End Sequence Position | Sequence |
| --- | --- | --- | --- | --- | --- | --- | --- |
| 1 | 386.1947 | 770.3748 | 770.4286 | -0.0538 | 421 | 428 | R.AVSVNPGK.- |
| 2 | 410.6379 | 819.2612 | 819.3181 | -0.0569 | 209 | 215 | K.CADSNPR.G |
| 3 | 413.6842 | 825.3538 | 825.4093 | -0.0555 | 371 | 377 | R.HSTTQPR.K |
| 4 | 429.1691 | 856.3236 | 856.3749 | -0.0513 | 101 | 107 | K.TFSVCSR.D |
| 5 | 430.1496 | 858.2846 | 858.3364 | -0.0518 | 81 | 86 | K.QMFTCR.V + Oxidation (M) |
| 6 | 502.2210 | 1002.4274 | 1002.4922 | -0.0649 | 381 | 389 | K.GSGFFVFSR.L |
| 8 | 517.2462 | 1032.4778 | 1032.5352 | -0.0574 | 249 | 257 | K.GTVNLTWSR.A |
| 9 | 729.2573 | 1456.5001 | 1456.5929 | -0.0928 | 290 | 300 | R.DWIEGETYQCR.V |
| 10 | 494.2233 | 1479.6479 | 1479.7430 | -0.0950 | 407 | 420 | R.AVHEAASPSQTVQR.A |
| 11 | 759.3623 | 1516.7101 | 1515.8256 | 0.8844 | 275 | 289 | R.NGTLTVTSTLPVGTR.D |
| 12 | 519.5506 | 1555.6299 | 1555.7267 | -0.0968 | 87 | 100 | R.VAHTPSSTDWVDNK.T |
| 13 | 801.3722 | 1600.7299 | 1600.8382 | -0.1083 | 234 | 248 | K.SPTITCLVVDLAPSK.G |
| 14 | 537.8715 | 1610.5927 | 1610.7035 | -0.1107 | 395 | 406 | R.AEWEQKDEFICR.A |
| 15 | 577.2753 | 1728.8040 | 1728.9331 | -0.1292 | 233 | 248 | R.KSPTITCLVVDLAPSK.G |
| 16 | 924.8891 | 1847.7636 | 1847.8842 | -0.1206 | 322 | 338 | R.AAPEVYAFATPEWPGSR.D |
| 18 | 722.9327 | 2165.7764 | 2165.9211 | -0.1448 | 190 | 207 | R.TYTCQVTYQGHTFEDSTK.K |
| 19 | 765.6268 | 2293.8585 | 2294.0161 | -0.1576 | 190 | 208 | R.TYTCQVTYQGHTFEDSTKK.C |

**Supplementary Table 9** Non-glycosylated IgE peptides identified in a chymotrypsin digest of IgE from a *PGM3* patient

| Match to: **IGHE_HUMAN** Score: **701** | | | | | | |  | |  | |  | |  |  | |  |  |  |
| --- | --- | --- | --- | --- | --- | --- | --- | --- | --- | --- | --- | --- | --- | --- | --- | --- | --- | --- |
| **RecName: Full=Ig epsilon chain C region** | | | | | | | | |  | |  | |  |  | |  |  |  |
| Sequence Coverage: **49%** | | | | | | |  | |  | |  | |  |  | |  |  |  |
| Fixed modifications: Carboxymethyl (C) | | | | | | | | |  | |  | |  |  | |  |  |  |
| Variable modifications: Oxidation (M) | | | | | | | | |  | |  | |  |  | |  |  |  |
| Cleavage by Chymotrypsin: cuts C-term side of FYWL unless next residue is P | | | | | | | | | | | | | |  | |  |  |  |
|  | | | |  |  | |  | |  | |  | |  |  | |  |  |  |
| Matched peptides shown in **Bold Red** | | | | | | | | | | |  | |  |  | |  |  |  |
| **1** ASTQSPSVFP LTRCCKNIPS NATSVTL**GCL ATGYFPEPVM VTW**DTGSLNG | | | | | | | | | | | | | | | |  |  |  |
| **51** TTMTLPATTL **TLSGHYATIS LLTVSGAWAK QMFTCRVAHT PSSTDWVDNK** | | | | | | | | | | | | | | | |  |  |  |
| **101 TFSVCSRDFT PPTVKILQSS CDGGGHFPPT IQLL**CLVSGY TPGTINITWL | | | | | | | | | | | | | | | |  |  |  |
| **151** EDGQVMDVDL STASTTQEGE LASTQSEL**TL SQKHWLSDRT YTCQVTY**QGH | | | | | | | | | | | | | | | |  |  |  |
| **201** TFEDSTKKCA DSNPRGVSAY **LSRPSPFDLF IRKSPTITCL VVDLAPSKGT** | | | | | | | | | | | | | | | |  |  |  |
| **251 VNLTW**SRASG KPVNHSTRKE EKQRNGTL**TV TSTLPVGTRD WIEGETY**QCR | | | | | | | | | | | | | | | |  |  |  |
| **301** VTHPHLPRAL MRSTTKTSGP RAAPEVY**AFA TPEWPGSRDK RTLACLIQNF** | | | | | | | | | | | | | | | |  |  |  |
| **351 MPEDISVQW**L HNEVQLPDAR HSTTQPRKTK GSGFF**VFSRL EVTRAEWEQK** | | | | | | | | | | | | | | | |  |  |  |
| **401 DEF**ICRAVHE AASPSQTVQR AVSVNPGK | | | | | | | | | | |  | |  |  | |  |  |  |
| Peptide Number | Observed | Calculated Theoretical Mass | Observed Mass (calculated) | | | Match Error Da | | Start Sequence Position | | End Sequence Position | | Sequence | | |  |  |  |  |
| 1 | 914.8584 | 1827.7022 | 1827.8211 | | | -0.1189 | | 28 | | 43 | | L.GCLATGYFPEPVMVTW.D | | |  |  |  |  |
| 2 | 922.8503 | 1843.686 | 1843.816 | | | -0.1300 | | 28 | | 43 | | L.GCLATGYFPEPVMVTW.D Oxidation (M) | | |  |  |  |  |
| 3 | 638.2904 | 1274.5663 | 1274.687 | | | -0.1207 | | 61 | | 72 | | L.TLSGHYATISLL.T | | |  |  |  |  |
| 4 | 531.2318 | 1060.4489 | 1060.5553 | | | -0.1064 | | 63 | | 72 | | L.SGHYATISLL.T | | |  |  |  |  |
| 5 | 309.1583 | 616.3021 | 616.3796 | | | -0.0775 | | 67 | | 72 | | Y.ATISLL.T | | |  |  |  |  |
| 6 | 613.2456 | 1224.4766 | 1224.5961 | | | -0.1195 | | 73 | | 83 | | L.TVSGAWAKQMF.T | | |  |  |  |  |
| 8 | 621.2469 | 1240.4792 | 1240.591 | | | -0.1118 | | 73 | | 83 | | L.TVSGAWAKQMF.T Oxidation (M) | | |  |  |  |  |
| 9 | 312.6213 | 623.228 | 623.3101 | | | -0.0821 | | 79 | | 83 | | W.AKQMF.T | | |  |  |  |  |
| 10 | 741.6179 | 2221.8319 | 2222.0062 | | | -0.1743 | | 84 | | 102 | | F.TCRVAHTPSSTDWVDNKTF.S | | |  |  |  |  |
| 11 | 436.1385 | 870.2624 | 870.3541 | | | -0.0917 | | 103 | | 109 | | F.SVCSRDF.T | | |  |  |  |  |
| 12 | 888.7308 | 2663.1707 | 2663.3628 | | | -0.1921 | | 110 | | 134 | | F.TPPTVKILQSSCDGGGHFPPTIQLL.C | | |  |  |  |  |
| 13 | 907.8568 | 1813.699 | 1813.8305 | | | -0.1315 | | 118 | | 134 | | L.QSSCDGGGHFPPTIQLL.C | | |  |  |  |  |
| 14 | 450.1888 | 898.3631 | 898.4661 | | | -0.1030 | | 179 | | 185 | | L.TLSQKHW.L | | |  |  |  |  |
| 15 | 343.1277 | 684.2409 | 684.3344 | | | -0.0935 | | 181 | | 185 | | L.SQKHW.L | | |  |  |  |  |
| 16 | 474.1898 | 1419.5477 | 1419.6895 | | | -0.1418 | | 181 | | 191 | | L.SQKHWLSDRTY.T | | |  |  |  |  |
| 18 | 377.6343 | 753.2541 | 753.3657 | | | -0.1116 | | 186 | | 191 | | W.LSDRTY.T | | |  |  |  |  |
| 19 | 386.6192 | 771.2239 | 771.3109 | | | -0.0870 | | 192 | | 197 | | Y.TCQVTY.Q | | |  |  |  |  |
| 20 | 589.7666 | 1177.5185 | 1177.6131 | | | -0.0946 | | 221 | | 230 | | Y.LSRPSPFDLF.I | | |  |  |  |  |
| 22 | 345.6397 | 689.2648 | 689.3497 | | | -0.0849 | | 222 | | 227 | | L.SRPSPF.D | | |  |  |  |  |
| 23 | 533.2238 | 1064.4331 | 1064.5291 | | | -0.0960 | | 222 | | 230 | | L.SRPSPFDLF.I | | |  |  |  |  |
| 24 | 397.1795 | 1188.5166 | 1188.6536 | | | -0.1370 | | 231 | | 240 | | F.IRKSPTITCL.V | | |  |  |  |  |
| 25 | 800.3767 | 1598.7388 | 1598.8668 | | | -0.1280 | | 241 | | 255 | | L.VVDLAPSKGTVNLTW.S | | |  |  |  |  |
| 27 | 1062.9504 | 2123.8862 | 2124.0375 | | | -0.1513 | | 279 | | 297 | | L.TVTSTLPVGTRDWIEGETY.Q | | |  |  |  |  |
| 28 | 726.3019 | 2175.884 | 2176.0735 | | | -0.1895 | | 328 | | 346 | | Y.AFATPEWPGSRDKRTLACL.I | | |  |  |  |  |
| 29 | 811.824 | 1621.6335 | 1621.7446 | | | -0.1111 | | 347 | | 359 | | L.IQNFMPEDISVQW.L Oxidation (M) | | |  |  |  |  |
| 30 | 311.1492 | 620.2839 | 620.3646 | | | -0.0807 | | 386 | | 390 | | F.VFSRL.E | | |  |  |  |  |
| 31 | 498.2185 | 1491.6338 | 1491.7834 | | | -0.1496 | | 386 | | 397 | | F.VFSRLEVTRAEW.E | | |  |  |  |  |
| 32 | 756.9823 | 2267.9252 | 2268.1174 | | | -0.1922 | | 386 | | 403 | | F.VFSRLEVTRAEWEQKDEF.I | | |  |  |  |  |
| 33 | 623.7736 | 1245.5327 | 1245.6465 | | | -0.1138 | | 388 | | 397 | | F.SRLEVTRAEW.E | | |  |  |  |  |
| 34 | 674.9405 | 2021.7997 | 2021.9806 | | | -0.1809 | | 388 | | 403 | | F.SRLEVTRAEWEQKDEF.I | | |  |  |  |  |

**Supplementary Table 10** Non-glycosylated IgE peptides identified in a trypsin digest of IgE from an atopic patient

| Match to: **gi\|119512** Score: **549** | | |  |  |  |  |  |
| --- | --- | --- | --- | --- | --- | --- | --- |
| **RecName: Full=Ig epsilon chain C region** | | | |  |  |  |  |
| Sequence Coverage: **29%** | | |  |  |  |  |  |
| Variable modifications: Oxidation (M),Carboxymethyl (C) | | | | |  |  |  |
| Cleavage by Trypsin: cuts C-term side of KR unless next residue is P | | | | | |  |  |
|  |  |  |  |  |  |  |  |
| Matched peptides shown in **Bold Red** | | | |  |  |  |  |
|  |  |  |  |  |  |  |  |
| **1** ASTQSPSVFP LTRCCKNIPS NATSVTLGCL ATGYFPEPVM VTWDTGSLNG | | | | | | | |
| **51** TTMTLPATTL TLSGHYATIS LLTVSGAWAK QMFTCRVAHT PSSTDWVDNK | | | | | | | |
| **101** **TFSVCSR**DFT PPTVKILQSS CDGGGHFPPT IQLLCLVSGY TPGTINITWL | | | | | | | |
| **151** EDGQVMDVDL STASTTQEGE LASTQSELTL SQKHWLSDR**T YTCQVTYQGH** | | | | | | | |
| **201 TFEDSTKKCA DSNPR**GVSAY LSRPSPFDLF IRKSPTITCL VVDLAPSKGT | | | | | | | |
| **251** VNLTWSR**ASG KPVNHSTR**KE EKQR**NGTLTV TSTLPVGTRD WIEGETYQCR** | | | | | | | |
| **301** VTHPHLPRAL MRSTTKTSGP R**AAPEVYAFA TPEWPGSR**DK RTLACLIQNF | | | | | | | |
| **351** MPEDISVQWL HNEVQLPDAR **HSTTQPR**KTK **GSGFFVFSR**L EVTRAEWEQK | | | | | | | |
| **401** DEFICR**AVHE AASPSQTVQR AVSVNPGK** | | | | | | | |
|  |  |  |  |  |  |  |  |

| Peptide Number | Observed | Calculated Theoretical Mass | Observed Mass (calculated) | Match Error Da | Start Sequence Position | End Sequence Position | Sequence |
| --- | --- | --- | --- | --- | --- | --- | --- |
| 1 | 386.2212 | 770.4279 | 770.4286 | -0.0007 | 101 | 107 | R.AVSVNPGK.- |
| 2 | 413.7123 | 825.4101 | 825.4093 | 0.0008 | 190 | 207 | R.HSTTQPR.K |
| 3 | 429.3000 | 856.5854 | 856.3749 | 0.2106 | 190 | 207 | K.TFSVCSR.D + Carboxymethyl (C) |
| 4 | 474.7163 | 947.4180 | 947.4131 | 0.0049 | 190 | 208 | K.KCADSNPR.G + Carboxymethyl (C) |
| 5 | 502.2601 | 1002.5057 | 1002.4922 | 0.0135 | 190 | 208 | K.GSGFFVFSR.L |
| 6 | 385.1000 | 1152.2782 | 1152.5999 | -0.3218 | 208 | 215 | R.ASGKPVNHSTR.K |
| 8 | 700.3000 | 1398.5854 | 1398.5874 | -0.0019 | 258 | 268 | R.DWIEGETYQCR.V |
| 9 | 729.3246 | 1456.6347 | 1456.5929 | 0.0418 | 275 | 289 | R.DWIEGETYQCR.V + Carboxymethyl (C) |
| 10 | 494.2518 | 1479.7336 | 1479.7430 | -0.0093 | 290 | 300 | R.AVHEAASPSQTVQR.A |
| 11 | 741.3728 | 1480.7310 | 1479.7430 | 0.9880 | 290 | 300 | R.AVHEAASPSQTVQR.A |
| 12 | 759.4183 | 1516.8221 | 1515.8256 | 0.9964 | 322 | 338 | R.NGTLTVTSTLPVGTR.D |
| 13 | 924.9681 | 1847.9216 | 1847.8842 | 0.0374 | 371 | 377 | R.AAPEVYAFATPEWPGSR.D |
| 14 | 703.6475 | 2107.9208 | 2107.9157 | 0.0051 | 381 | 389 | R.TYTCQVTYQGHTFEDSTK.K |
| 15 | 722.9897 | 2165.9472 | 2165.9211 | 0.0261 | 407 | 420 | R.TYTCQVTYQGHTFEDSTK.K + Carboxymethyl (C) |
| 16 | 746.3645 | 2236.0717 | 2236.0106 | 0.0611 | 407 | 420 | R.TYTCQVTYQGHTFEDSTKK.C |
| 17 | 765.6843 | 2294.0311 | 2294.0161 | 0.0150 | 421 | 428 | R.TYTCQVTYQGHTFEDSTKK.C + Carboxymethyl (C) |

**Supplementary Table 11** Non-glycosylated IgE peptides identified in a chymotrypsin digest of IgE from an atopic patient

| Match to: **IGHE_HUMAN** Score: **830** | | |  |  |  |  |  |
| --- | --- | --- | --- | --- | --- | --- | --- |
| **RecName: Full=Ig epsilon chain C region** | | | |  |  |  |  |
| Sequence Coverage: **52%** | | |  |  |  |  |  |
| Fixed modifications: Carboxymethyl (C) | | | |  |  |  |  |
| Variable modifications: Oxidation (M) | | | |  |  |  |  |
| Cleavage by Chymotrypsin: cuts C-term side of FYWL unless next residue is P | | | | | | |  |
|  |  |  |  |  |  |  |  |
| Matched peptides shown in **Bold Red** | | | | |  |  |  |
|  |  |  |  |  |  |  |  |
| **1** ASTQSPSVFP LTRCCKNIPS NATSVTL**GCL ATGYFPEPVM VTW**DTGSLNG | | | | | | | |
| **51** TTMTLPATTL **TLSGHYATIS LLTVSGAWAK QMFTCRVAHT PSSTDWVDNK** | | | | | | | |
| **101 TFSVCSRDFT PPTVKILQSS CDGGGHFPPT IQLL**CLVSGY TPGTINITWL | | | | | | | |
| **151** EDGQVMDVDL STASTTQEGE LASTQSELTL SQKHW**LSDRT YTCQVTY**QGH | | | | | | | |
| **201** TFEDSTKKCA DSNPRGVSAY **LSRPSPFDLF IRKSPTITCL VVDLAPSKGT** | | | | | | | |
| **251 VNLTW**SRASG KPVNHSTRKE EKQRNGTL**TV TSTLPVGTRD WIEGETY**QCR | | | | | | | |
| **301** VTHPHLPRAL **MRSTTKTSGP RAAPEVYAFA TPEWPGSRDK RTLACLIQNF** | | | | | | | |
| **351 MPEDISVQW**L HNEVQLPDAR HSTTQPRKTK GSGFF**VFSRL EVTRAEWEQK** | | | | | | | |
| **401 DEF**ICRAVHE AASPSQTVQR AVSVNPGK | | | | | | | |

| Peptide Number | Observed | Calculated Theoretical Mass | Observed Mass (calculated) | Match Error Da | Start Sequence Position | End Sequence Position | Sequence |
| --- | --- | --- | --- | --- | --- | --- | --- |
| 1 | 914.8603 | 1827.7061 | 1827.8211 | -0.1150 | 28 | 43 | L.GCLATGYFPEPVMVTW.D |
| 3 | 922.857 | 1843.6994 | 1843.816 | -0.1166 | 28 | 43 | L.GCLATGYFPEPVMVTW.D Oxidation (M) |
| 4 | 757.301 | 1512.5875 | 1512.6959 | -0.1084 | 31 | 43 | L.ATGYFPEPVMVTW.D Oxidation (M) |
| 5 | 339.1257 | 676.2369 | 676.318 | -0.0811 | 61 | 66 | L.TLSGHY.A |
| 6 | 638.3035 | 1274.5924 | 1274.687 | -0.0946 | 61 | 72 | L.TLSGHYATISLL.T |
| 8 | 531.2373 | 1060.4601 | 1060.5553 | -0.0952 | 63 | 72 | L.SGHYATISLL.T |
| 9 | 613.2565 | 1224.4984 | 1224.5961 | -0.0977 | 73 | 83 | L.TVSGAWAKQMF.T |
| 10 | 621.2522 | 1240.4897 | 1240.591 | -0.1013 | 73 | 83 | L.TVSGAWAKQMF.T Oxidation (M) |
| 11 | 312.6236 | 623.2325 | 623.3101 | -0.0776 | 79 | 83 | W.AKQMF.T |
| 12 | 741.6309 | 2221.8707 | 2222.0062 | -0.1355 | 84 | 102 | F.TCRVAHTPSSTDWVDNKTF.S |
| 13 | 436.1456 | 870.2766 | 870.3541 | -0.0775 | 103 | 109 | F.SVCSRDF.T |
| 14 | 574.2549 | 1719.7428 | 1719.8865 | -0.1437 | 103 | 117 | F.SVCSRDFTPPTVKIL.Q |
| 15 | 434.7377 | 867.4608 | 867.5429 | -0.0821 | 110 | 117 | F.TPPTVKIL.Q |
| 16 | 888.738 | 2663.1923 | 2663.3628 | -0.1705 | 110 | 134 | F.TPPTVKILQSSCDGGGHFPPTIQLL.C |
| 18 | 907.8614 | 1813.7082 | 1813.8305 | -0.1223 | 118 | 134 | L.QSSCDGGGHFPPTIQLL.C |
| 19 | 377.635 | 753.2554 | 753.3657 | -0.1103 | 186 | 191 | W.LSDRTY.T |
| 20 | 386.6259 | 771.2372 | 771.3109 | -0.0737 | 192 | 197 | Y.TCQVTY.Q |
| 24 | 402.3095 | 802.6044 | 802.4337 | 0.1707 | 221 | 227 | Y.LSRPSPF.D |
| 27 | 516.2301 | 1030.4456 | 1030.5447 | -0.0991 | 221 | 229 | Y.LSRPSPFDL.F |
| 28 | 589.7648 | 1177.515 | 1177.6131 | -0.0981 | 221 | 230 | Y.LSRPSPFDLF.I |
| 31 | 533.2232 | 1064.4318 | 1064.5291 | -0.0973 | 222 | 230 | L.SRPSPFDLF.I |
| 32 | 595.2869 | 1188.5593 | 1188.6536 | -0.0943 | 231 | 240 | F.IRKSPTITCL.V |
| 33 | 656.8281 | 1311.6416 | 1311.7398 | -0.0982 | 241 | 253 | L.VVDLAPSKGTVNL.T |
| 34 | 800.3842 | 1598.7539 | 1598.8668 | -0.1129 | 241 | 255 | L.VVDLAPSKGTVNLTW.S |
| 35 | 708.9669 | 2123.8789 | 2124.0375 | -0.1586 | 279 | 297 | L.TVTSTLPVGTRDWIEGETY.Q |
| 36 | 623.2636 | 1866.7689 | 1866.9258 | -0.1569 | 311 | 327 | L.MRSTTKTSGPRAAPEVY.A Oxidation (M) |
| 37 | 726.3089 | 2175.9049 | 2176.0735 | -0.1686 | 328 | 346 | Y.AFATPEWPGSRDKRTLACL.I |
| 38 | 984.3832 | 1966.7518 | 1966.8804 | -0.1286 | 344 | 359 | L.ACLIQNFMPEDISVQW.L Oxidation (M) |
| 40 | 803.9591 | 1605.9037 | 1605.7497 | 0.1540 | 347 | 359 | L.IQNFMPEDISVQW.L |
| 41 | 811.8167 | 1621.6189 | 1621.7446 | -0.1257 | 347 | 359 | L.IQNFMPEDISVQW.L Oxidation (M) |
| 42 | 311.1542 | 620.2938 | 620.3646 | -0.0708 | 386 | 390 | F.VFSRL.E |
| 43 | 498.2216 | 1491.643 | 1491.7834 | -0.1404 | 386 | 397 | F.VFSRLEVTRAEW.E |
| 44 | 756.9937 | 2267.9594 | 2268.1174 | -0.1580 | 386 | 403 | F.VFSRLEVTRAEWEQKDEF.I |
| 45 | 416.1805 | 1245.5196 | 1245.6465 | -0.1269 | 388 | 397 | F.SRLEVTRAEW.E |
| 46 | 674.949 | 2021.8251 | 2021.9806 | -0.1555 | 388 | 403 | F.SRLEVTRAEWEQKDEF.I |
